# Supplementary material for: Prospective cohort study of exposure to tobacco imagery in popular films and smoking uptake among children in southern India
Source: PLoS One. 2021 Aug 5;16(8):e0253593. doi: 10.1371/journal.pone.0253593 (PMC8341541; doi:10.1371/journal.pone.0253593)
Supplement: S1 File — (ZIP) [file pone.0253593.s002.zip › Year_One_Questionneire_English.pdf]

Preventing smoking uptake among adolescents – A primary prevention initiative for Chronic Lung Diseases in India.

QUESTIONNAIRE - Year One

1. OMR Serial No.	2. School Code	3. Enrollment Number	4. Date of Birth
	0–9 (6 digits)	0–9 (9 digits)	D D / M M / 2 0 Y Y

5. Gender	6. Class	7. Section	8. Religion	9. Father's Education	10. Mother's Education
Male	6	A	Hindu	Illiterate	Illiterate
Female	7	B	Christian	Can Read	Can Read
	8	C	Muslim	1-4 Std	1-4 Std
		D	Jain	5-7 Std	5-7 Std
		E	Other	8-10 Std	8-10 Std
		F		11-12 Std	11-12 Std
		G		Diploma	Diploma
		H		Graduate	Graduate
				Post-Graduate	Post-Graduate
				Professional	Professional

12. Father's Occupation		13. Mother's Occupation	

Note: A tobacco product is a substance that contains tobacco. There are two types of products:

Smoking Tobacco : Beedis, Cigarettes, Cigars, Cheroots, Rolled Cigarettes, any tobacco rolled in maize leaf or newspaper/ paper, Hukkha, Pipes, Chillum, Chutta.

Smokeless Tobacco : Tobacco leaf, Betel quid with tobacco, Sada/Surti, Khaini or Tobacco Lime Mixture, Gutkha, Pan Masala with Zarda, Gul, Gudaku, Mishri

There are some questions which ask specifically about smoking or smokeless tobacco use, while some of them includes both forms and ask about tobacco in general. Please read the questions carefully and answer them. If you have any doubts please feel free to ask the project staff and clarify.

1. THESE QUESTIONS ARE REGARDING AWARENESS ABOUT EFFECTS OF TOBACCO USE

1.1 What do you think is the effect of tobacco use on one's health?
- Ⓐ Good
- Ⓑ Bad
- Ⓒ Not sure

1.2 Does tobacco use by a young person harm his/her health immediately?
- Ⓐ Yes
- Ⓑ No
- Ⓒ Not sure

1.3 Do you think it is safe to smoke or chew tobacco for only one to two years?
- Ⓐ Yes
- Ⓑ No
- Ⓒ Not sure

1.4 Does it harm your health if you are near a person who is smoking?
- Ⓐ Yes
- Ⓑ No
- Ⓒ Not sure

1.5 Would stopping tobacco use improve a person's health?
- Ⓐ Yes
- Ⓑ No
- Ⓒ Not sure

2. THESE QUESTIONS ARE ABOUT ANTI-TOBACCO POLICIES

2.1 Is there a law which stops people from smoking in public places?
- Ⓐ Yes
- Ⓑ No
- Ⓒ Don't know

2.2 Is there a law which bans people from selling smokeless tobacco (eg.gutkha) in our state?
- Ⓐ Yes
- Ⓑ No
- Ⓒ Don't know

2.3 Is there a law which prohibits tobacco advertising on television channels and print media?
- Ⓐ Yes
- Ⓑ No
- Ⓒ Don't know

2.4 Is there a display of “No smoking area - smoking here is an offence” board inside your school”?
- Ⓐ Yes
- Ⓑ No
- Ⓒ Not sure

2.5 Is there a display of “Tobacco Free School” or “Tobacco Free Institution” board at a prominent place on the boundary wall outside the main entrance of your school?
- Ⓐ Yes
- Ⓑ No
- Ⓒ Not sure

2.6 Have you observed any picture and/or written warning on a tobacco pack about its harmful effects?
- Ⓐ Yes
- Ⓑ No
- Ⓒ I have not seen any tobacco pack

2.7 Have you observed any advertisement board encouraging tobacco use near the school premises?
- Ⓐ Yes
- Ⓑ No

2.8 During the last 30 days have you observed any free distribution of tobacco products around your school?
- Ⓐ Yes
- Ⓑ No

2.9 During the last 30 days, have you observed any of the tobacco products being sold within a distance of 100 yards (nearly 90 metres) from your school?
- Ⓐ Yes
- Ⓑ No

3. THE NEXT QUESTIONS ARE ABOUT FILMS AND MUSIC VIDEOS

(Darken all the circles which apply to you)

3.1 Which of these Kannada movies have you seen?
- Ⓐ Kirik Party
- Ⓑ Master Piece
- Ⓒ Godi Banna Sadarana Maikattu
- Ⓓ Jaggu Dada
- Ⓔ Rangitaranga
- Ⓕ Ranavikrama
- Ⓖ Virat
- Ⓗ Ranna
- Ⓘ Airavatha
- Ⓙ Kotigobba-2
- Ⓚ Not seen any of these movies

3.2 Which of these Hindi movies have you seen?
- Ⓐ Detective Byomkesh Bakshy
- Ⓑ Kabali
- Ⓒ Rustom
- Ⓓ Welcome Back
- Ⓔ Shivay
- Ⓕ Baby
- Ⓖ Ae Dil Hai Mushkil
- Ⓗ Prem Rathan Dhan Payo
- Ⓘ Not seen any of these movies

3.3 Which of these Tulu movies have you seen?
- Ⓐ Dabak Daba Aisa
- Ⓑ Chandikori
- Ⓒ Yakka Saka
- Ⓓ Not seen any of these movies

3.4 Which of these other language movies have you seen?
- Ⓐ Kabali (Tamil)
- Ⓑ I (Tamil)
- Ⓒ Theri (Tamil)
- Ⓓ Pulimuragan (Malyalam)
- Ⓔ Srimanthudu (Telugu)
- Ⓕ Logan (English)
- Ⓖ Not seen any of these movies

3.5 Which of these Hindi music videos have you seen?
- Ⓐ Bolna mahi bolna
- Ⓑ Dheere dheere se
- Ⓒ Hamari Adhuri Kahani
- Ⓓ Jeena jeena
- Ⓔ Not seen any of these music videos

3.6 Which of these Kannada music videos have you seen?
- Ⓐ Ne muddada
- Ⓑ Hudugi Kannu
- Ⓒ Not seen any of these music videos

3.7 Have you noticed any anti-smoking messages while watching any of the above movies/ music videos?
- Ⓐ Yes
- Ⓑ No

3.8 Have you noticed any actor/actress smoking in the movies/ music videos which you have seen?
- Ⓐ Yes
- Ⓑ No

3.9 Have you noticed any actor/actress using smokeless tobacco in the movies/ music videos which you have seen?
- Ⓐ Yes
- Ⓑ No

4. THESE QUESTIONS ARE ABOUT SHOPS

4.1 How often do you notice tobacco products on display when you go to supermarkets?
- Ⓐ Every time
- Ⓑ Most times
- Ⓒ Sometimes
- Ⓓ Hardly ever
- Ⓔ Never
- Ⓕ I don't go to supermarket

4.2 How often do you notice tobacco products on display when you go to small shops (e.g. Small grocery shops, pan shop)?
- Ⓐ Every time
- Ⓑ Most times
- Ⓒ Sometimes
- Ⓓ Hardly ever
- Ⓔ Never
- Ⓕ I don't go to small shops

4.3 When you go to supermarkets or small shops have you noticed any tobacco brands on display?
- Ⓐ Yes, I have noticed

  If yes, mention the brand..

  ....
- Ⓑ No, I have never noticed any brands on display
- Ⓒ I don't remember the brands
- Ⓓ I don't go to shops/supermarkets

4.4 If anyone of your age tried to purchase tobacco product in any shop, do you think they will get it?
- Ⓐ Yes
- Ⓑ No
- Ⓒ Don't know

5. THESE QUESTIONS ARE ABOUT SMOKING

5.1 Is smoking allowed in your home?
- Ⓐ Yes
- Ⓑ No

5.2 Does anybody in your family smoke? (Darken all the circles that apply to you)
- Ⓐ None
- Ⓑ Mother
- Ⓒ Father
- Ⓓ Brother
- Ⓔ Sister
- Ⓕ Others

5.3 How many of your friends smoke?
- Ⓐ None
- Ⓑ One
- Ⓒ Two
- Ⓓ Three or more
- Ⓔ Not sure

5.4 Have you seen anyone smoke inside your school building or school compound?
- Ⓐ Yes
- Ⓑ No

5.5 Please read the options carefully and mark one option which applies to you regarding smoking:
- Ⓐ I have never smoked
- Ⓑ I have smoked in the past but not in the last 30 days
- Ⓒ I smoke sometimes but less than once a week
- Ⓓ I smoke one to six times a week
- Ⓔ I smoke more than six times a week

5.6 What was your age when you first tried smoking?
- Ⓐ I have never tried
- Ⓑ 7 years old or less
- Ⓒ 8 years old
- Ⓓ 9 years old
- Ⓔ 10 years old
- Ⓕ 11 years old
- Ⓖ 12 years old
- Ⓗ 13 years old
- Ⓘ 14 years old
- Ⓙ 15 years old or more

5.7 What did you smoke for the first time?
- Ⓐ I have not smoked at all
- Ⓑ I smoked cigarettes
- Ⓒ I smoked beedis
- Ⓓ I smoked hukkha
- Ⓔ If any other, please specify:____________________

5.8 What was the main reason for you to start smoking?
- Ⓐ I have never smoked
- Ⓑ Feeling alone
- Ⓒ Friend's pressure
- Ⓓ Picked up from elders
- Ⓔ Curiosity
- Ⓕ Feel grown up
- Ⓖ Tension
- Ⓗ To have more friends
- Ⓘ To look Stylish
- Ⓙ Actor/s smoking
- Ⓚ Other: If other, please specify:____________________

5.9 How did you get your cigarettes/beedis when you smoked the first time?
- (A) I have never smoked
- (B) I bought it from a shop
- (C) I bought it on the Internet/online
- (D) I gave someone else money to buy it for me
- (E) I borrowed it from someone else
- (F) I got it from Family member
- (G) I smoked a half burnt cigarette/ beedi
- (H) If others, please specify: ______________________________

6. THESE QUESTIONS ARE ABOUT SMOKING IN THE PAST ONE MONTH (30 days)

6.1 How many times did you smoke in the last 30 days?
- (A) I have never smoked
- (B) Less than once a week
- (C) One to three times a week
- (D) Four to six times a week
- (E) More than six times a week

6.2 During the last 30 days (one month), how many cigarettes did you buy for yourself?
- (A) None
- (B) One cigarette
- (C) 2-9 cigarettes
- (D) A pack of 10 cigarettes
- (E) A pack of 20 cigarettes
- (F) More than 20 cigarettes

6.3 How much did you spend on your cigarettes in the past 30 days for yourself?
- (A) None
- (B) I smoked but didn't buy
- (C) Less than Rs10
- (D) Rs.11 to 30
- (E) Rs.31 to 60
- (F) Rs 61 to 100
- (G) More than Rs100

6.4 During the past 30 days, how many beedis did you buy for yourself?
- (A) None
- (B) One beedi
- (C) 2-5 beedis
- (D) 6-10 beedis
- (E) 11 -20 beedis
- (F) A pack of 25 beedis

6.5 How much did you spend on your beedis that you smoked in the past 30 days?
- (A) None
- (B) I smoked but didn't buy
- (C) Less than Rs10
- (D) Rs 11- 20
- (E) Rs 21 – 30
- (F) More than Rs 30

6.6 How many cigarettes have you smoked in the past 7 days?
- (A) None
- (B) 1 to 2
- (C) 3 to 5
- (D) 6 or more

6.7 How many beedis have you smoked in the past 7 days?
- (A) None
- (B) 1 to 2
- (C) 3 to 5
- (D) 6 or more

7. NEXT QUESTIONS ASK ABOUT YOUR PLANS AND THOUGHTS TO TRY SMOKING

7.1 Do you think that you will try smoking soon?
- (A) Yes
- (B) No

7.2 If one of your best friends were to offer you to smoke, would you try it?
- (A) Definitely yes
- (B) Probably yes
- (C) Probably not
- (D) Definitely not

7.3 Do you think you will smoke any time during the next one year?
- (A) Definitely yes
- (B) Probably yes
- (C) Probably not
- (D) Definitely not

7.4 Do you think you will smoke any time once you go to college?
- (A) Definitely yes
- (B) Probably yes
- (C) Probably not
- (D) Definitely not

8. THE NEXT SET OF QUESTIONS ARE ABOUT SMOKELESS TOBACCO USE (chewable tobacco, gutkha, khaini, zarda, snuff)

8.1 Is using smokeless tobacco allowed in your home?
- (A) Yes
- (B) No

8.2 Does anybody in your family use smokeless tobacco? (Darken all the circles which applies to you)
- (A) None
- (B) Mother
- (C) Father
- (D) Brother
- (E) Sister
- (F) Others

8.3 How many of your friends use smokeless tobacco?
- (A) None
- (B) One
- (C) Two
- (D) Three or more
- (E) Not sure

8.4 Please read the options carefully and mark one option which applies to you regarding use of smokeless tobacco:
- (A) I have never used smokeless tobacco
- (B) I have used smokeless tobacco in the past but not in the last 30 days
- (C) I use smokeless tobacco sometimes but less than once a week
- (D) I use smokeless tobacco one to six times a week
- (E) I use smokeless tobacco more than six times a week

8.5 What was your age when you first tried smokeless tobacco?
- (A) I have never tried
- (B) 7 years old or less
- (C) 8 years old
- (D) 9 years old
- (E) 10 years old
- (F) 11 years old
- (G) 12 years old
- (H) 13 years old
- (I) 14 years old
- (J) 15 years old or more

8.6 What was the main reason for you to start using smokeless tobacco?
- (A) I have never used
- (B) Feeling alone
- (C) Friends pressure
- (D) Picked up from elders
- (E) Curiosity
- (F) Feel grown up
- (G) Tension
- (H) To have more friends
- (I) To know the taste
- (J) To look Stylish
- (K) Other

If other, please specify:______________________________

8.7 In last 30 days, which of the following smokeless tobacco products have you used?(Darken all the circles that apply to you)
- (A) I have never used
- (B) Snuff
- (C) Khaini
- (D) Chewable tobacco
- (E) Gutkha
- (F) Zarda
- (G) Others

If others, please specify: ______________________________

8.8 During the past 30 days (one month), how many packets of smokeless tobacco did you buy for yourself?
- (A) None, as I have never used
- (B) I used but didn't buy
- (C) One packet
- (D) 2-5 packets
- (E) 5 - 10 packets
- (F) More than 10 packets

8.9 How many smokeless tobacco packets have you chewed in the last 7 days?
- (A) None
- (B) One packet
- (C) 2-5 packets
- (D) 6 - 10 packets
- (E) More than 10 packets

9. THE NEXT QUESTIONS ASK ABOUT YOUR PLANS AND THOUGHTS ABOUT USING SMOKELESS TOBACCO

9.1 Do you think that you will try smokeless tobacco soon?
- (A) Yes
- (B) No

9.2 If one of your best friends were to offer you any kind of smokeless tobacco, would you try it?
- (A) Definitely yes
- (B) Probably yes
- (C) Probably not
- (D) Definitely not

9.3 Do you think you will use smokeless tobacco at any time during the next one year?

Ⓐ Definitely yes Ⓑ Probably yes
Ⓒ Probably not Ⓓ Definitely not

9.4 Do you think you will use smokeless tobacco once you go to college?

Ⓐ Definitely yes Ⓑ Probably yes
Ⓒ Probably not Ⓓ Definitely not

10. THESE ARE SOME QUESTIONS WHICH DEAL WITH STOPPING TOBACCO USE

10.1 Have you ever felt like stopping tobacco use?

Ⓐ Yes Ⓑ No
Ⓒ I have never used tobacco

10.2 Have you tried stopping tobacco use?

Ⓐ Yes Ⓑ No
Ⓒ I have never used tobacco

11. THE NEXT QUESTIONS ARE ABOUT AWARENESS OF ANTI-TOBACCO ACTIVITIES

11.1 Was any educational class taken in your school about health hazards of tobacco and tobacco products in the past one year?

Ⓐ Yes Ⓑ No
Ⓒ Not Sure

11.2 Were you involved in any anti-tobacco activities in the past one year?

Ⓐ Yes
Ⓑ No

11.3 How many anti-tobacco messages have you heard/ seen on radio/TV in the past 30 days?

Ⓐ None Ⓑ One to five messages
Ⓒ Six to ten messages Ⓓ More than ten messages

11.4 How many anti-tobacco posters have you seen in the past 30 days?

Ⓐ None Ⓑ One to five messages
Ⓒ Six to ten messages Ⓓ More than ten messages

11.5 How many anti-tobacco messages have you seen in newspapers / magazines in the past 30 days?

Ⓐ None Ⓑ One to five messages
Ⓒ Six to ten messages Ⓓ More than ten messages
Ⓔ I don't read newspapers/ magazine

12. THESE QUESTIONS ARE ABOUT TOBACCO ADVERTISEMENTS

12.1 In the last 30 days, have you noticed any tobacco product advertisements in/on Public transport vehicles (e.g. buses, trains, taxis etc)

Ⓐ Yes
Ⓑ No

12.2 In the last 30 days, have you noticed any tobacco product advertisements on walls in public places?

Ⓐ Yes
Ⓑ No

12.3 In the last 30 days, have you noticed any tobacco product advertisements on Internet?

Ⓐ Yes
Ⓑ No

12.4 In the last 30 days, have you heard/seen any tobacco advertisements on radio/TV?

Ⓐ Yes
Ⓑ No

12.5 In the last 30 days, have you noticed any tobacco product advertisements in Newspapers/Magazines?

Ⓐ Yes
Ⓑ No

13. PLEASE READ THE FOLLOWING SENTENCES AND TELL US HOW THEY DESCRIBE YOURSELF

13.1 I ignore rules that get in the way of what I want to do

Ⓐ Never
Ⓑ Sometimes
Ⓒ Often

13.2 I do things my parents wouldn't want me to do

Ⓐ Never
Ⓑ Sometimes
Ⓒ Often

13.3 I get into trouble with authorities at school, work, or other places

Ⓐ Never
Ⓑ Sometimes
Ⓒ Often

14. THESE QUESTIONS ARE ABOUT YOURSELF AND YOUR PERFORMANCE IN EXAMS

14.1 Please read the following sentence and mark the option that best suits you. "I think I have high self-esteem".

Ⓐ Strongly agree
Ⓑ Agree
Ⓒ Neither agree nor disagree
Ⓓ Disagree
Ⓔ Strongly disagree

14.2 How would you describe your performance in the last annual examination?

Ⓐ Excellent Ⓑ Good
Ⓒ Average Ⓓ Below Average

15. THE LAST QUESTION IS ABOUT YOUR HOUSE

15.1 Mark all the following items which are there in your house or belongs to any person who lives in your house:

Ⓐ Electricity Ⓑ Toilet with a flush
Ⓒ Car Ⓓ Moped/scooter/motorcycle
Ⓔ Television Ⓕ Refrigerator
Ⓖ Washing machine Ⓗ Fixed telephone
Ⓘ Mobile phone Ⓙ Radio

Thank You for Completing this Questionnaire

Signature of Research Assistant
