# Supplementary material for: Prospective cohort study of exposure to tobacco imagery in popular films and smoking uptake among children in southern India
Source: PLoS One. 2021 Aug 5;16(8):e0253593. doi: 10.1371/journal.pone.0253593 (PMC8341541; doi:10.1371/journal.pone.0253593)
Supplement: S1 File — (ZIP) [file pone.0253593.s002.zip › Year_One_Questionneire_Kannada.pdf]

ಹದಿಹರೆಯದವರಲ್ಲಿ ಧೂಮಪಾನ ಸೇವನೆಯ ನಿಯಂತ್ರಣ–ಭಾರತದಲ್ಲಿ ದೀರ್ಘಕಾಲೀನ ಶ್ವಾಸಕೋಶದ ಖಾಯಿಲೆಯನ್ನು ತಡೆಗಟ್ಟುವ ಪ್ರಕ್ರಿಯೆಯ ಪ್ರಾಥಮಿಕ ಹಂತ

ಪ್ರಶ್ನಾವಳಿ – ಮೊದಲ ವರ್ಷ

1. ಓ.ಎಮ್.ಆರ್ ಕ್ರಮ ಸಂಖ್ಯೆ	2. ಶಾಲಾ ಕೋಡ್	3. ಹಾಜರಿ ಸಂಖ್ಯೆ	4. ಹುಟ್ಟಿದ ದಿನಾಂಕ
	0–9 (6 ಅಂಕಿಗಳು)	0–9 (9 ಅಂಕಿಗಳು)	D D / M M / 2 0 Y Y

5. ಲಿಂಗ	6. ತರಗತಿ	7. ವಿಭಾಗ	8. ಧರ್ಮ	9. ತಂದೆಯ ಶಿಕ್ಷಣ	10. ತಾಯಿಯ ಶಿಕ್ಷಣ
ಗಂಡು ◯	6 ◯	A ◯	ಹಿಂದೂ ◯	ಅನಕ್ಷರಸ್ಥ ◯	ಅನಕ್ಷರಸ್ಥ ◯
ಹೆಣ್ಣು ◯	7 ◯	B ◯	ಕ್ರಿಶ್ಚಿಯನ್ ◯	ಓದಲು ಮಾತ್ರ ◯	ಓದಲು ಮಾತ್ರ ◯
	8 ◯	C ◯	ಮುಸ್ಲಿಂ ◯	1-4 ◯	1-4 ◯
		D ◯	ಜೈನ್ ◯	5-7 ◯	5-7 ◯
		E ◯	ಇತರೆ ◯	8-10 ◯	8-10 ◯
		F ◯		11-12 ◯	11-12 ◯
		G ◯		ಡಿಪ್ಲೋಮ ◯	ಡಿಪ್ಲೋಮ ◯
		H ◯		ಪದವಿ ◯	ಪದವಿ ◯
				ಸ್ನಾತಕೋತ್ತರ ◯	ಸ್ನಾತಕೋತ್ತರ ◯
				ವೃತ್ತಿಪರ ◯	ವೃತ್ತಿಪರ ◯

12. ತಂದೆಯ ಉದ್ಯೋಗ		13. ತಾಯಿಯ ಉದ್ಯೋಗ	

ಗಮನಿಸಿ : ತಂಬಾಕು ಉತ್ಪನ್ನವು ತಂಬಾಕುವನ್ನೊಳಗೊಂಡ ವ್ಯಸನಕಾರಕ ವಸ್ತುವಾಗಿದೆ ಮತ್ತು ಇದರಲ್ಲಿ 2 ವಿಧದ ಉತ್ಪನ್ನಗಳಿವೆ :

ಹೊಗೆಸಹಿತ ತಂಬಾಕು – ಬೀಡಿ, ಸಿಗರೇಟ್, ಸಿಗಾರ್, ಚಿರೂಟ್ಸ್, ಸುರುಳಿ ಸಿಗರೇಟ್‌ಗಳು, ಯಾವುದೇ ತಂಬಾಕು ಮೆಕ್ಕೆಜೋಳದ ಎಲೆ ಅಥವಾ ವಾರ್ತಾ ಪತ್ರಿಕೆಯಲ್ಲಿ ಸುತ್ತಿಕೊಂಡಿರುವುದು, ಹುಕ್ಕಾ, ಪೈಪ್‌ಗಳು, ಚಿಲ್ಲಮ್, ಚುಟ್ಟಾ

ಹೊಗೆರಹಿತ ತಂಬಾಕು – ತಂಬಾಕು ಎಲೆ, ಅಡಿಕೆ ತುಂಡಿನ ಜೊತೆ ತಂಬಾಕು, ಸಾಡಾ/ಸುರ್ತಿ, ಖೈನಿ ಅಥವಾ ತಂಬಾಕು ಹುಳಿ ಮಿಶ್ರಣ, ಗುಟ್ಕ, ಜರ್ದಾ ಜೊತೆ ಪಾನ್ ಮಸಾಲ, ಗುಲ್, ಗುಡಾಕು, ಮಿಶ್ರಿ

ಕೆಲವು ಪ್ರಶ್ನೆಗಳು ಮುಖ್ಯವಾಗಿ ಹೊಗೆಸಹಿತ ತಂಬಾಕು ಅಥವಾ ಹೊಗೆರಹಿತ ತಂಬಾಕು ಉತ್ಪನ್ನದ ಬಗ್ಗೆ ಆಗಿವೆ. ಕೆಲವು ಪ್ರಶ್ನೆಗಳು 2 ವಿಧದ ತಂಬಾಕಿನ ಬಗ್ಗೆ ಸಾಮಾನ್ಯ ಪ್ರಶ್ನೆಗಳಾಗಿವೆ. ಪ್ರಶ್ನೆಗಳನ್ನು ಸರಿಯಾಗಿ ಓದಿ ಉತ್ತರಿಸಿ. ಸಂಶಯಗಳಿದ್ದರೆ ಸಿಬ್ಬಂದಿಗಳಲ್ಲಿ ಕೇಳಿ ಪರಿಹರಿಸಿಕೊಳ್ಳಿ.

1. ಈ ಪ್ರಶ್ನೆಗಳು ತಂಬಾಕಿನ ಪರಿಣಾಮಗಳ ತಿಳುವಳಿಕೆಯ ಬಗ್ಗೆ ಆಗಿವೆ

1.1 ತಂಬಾಕು ಬಳಕೆಯಿಂದ ಒಬ್ಬ ವ್ಯಕ್ತಿಯ ಆರೋಗ್ಯದ ಮೇಲೆ ಯಾವ ರೀತಿಯ ಪರಿಣಾಮ ಬೀರುತ್ತದೆ?
- Ⓐ ಉತ್ತಮ
- Ⓑ ಕೆಟ್ಟ ಪರಿಣಾಮ
- Ⓒ ಸರಿಯಾಗಿ ತಿಳಿದಿಲ್ಲ

1.2 ಯುವಜನತೆ ತಂಬಾಕಿನ ಬಳಕೆ ಮಾಡಿದರೆ ಆರೋಗ್ಯದ ಮೇಲೆ ಬಹು ಬೇಗನೆ ಕೆಟ್ಟ ಪರಿಣಾಮ ಬೀರುತ್ತದೆಯೇ?
- Ⓐ ಹೌದು
- Ⓑ ಇಲ್ಲ
- Ⓒ ಸರಿಯಾಗಿ ತಿಳಿದಿಲ್ಲ

1.3 ಕೇವಲ ಒಂದು ಅಥವಾ ಎರಡು ವರ್ಷಕ್ಕೆ ಮಾತ್ರ ಧೂಮಪಾನ ಅಥವಾ ತಂಬಾಕು ಸೇವನೆ ಮಾಡಿದರೆ ಸುರಕ್ಷಿತವೇ?
- Ⓐ ಹೌದು
- Ⓑ ಇಲ್ಲ
- Ⓒ ಸರಿಯಾಗಿ ತಿಳಿದಿಲ್ಲ

1.4 ನಿಮ್ಮ ಸುತ್ತಮುತ್ತಲಿನಲ್ಲಿ ಯಾರಾದರೂ ಧೂಮಪಾನ ಮಾಡಿದರೆ ಅದು ನಿಮ್ಮ ಆರೋಗ್ಯಕ್ಕೆ ಹಾನಿ ಉಂಟುಮಾಡುತ್ತದೆಯೇ?
- Ⓐ ಹೌದು
- Ⓑ ಇಲ್ಲ
- Ⓒ ಸರಿಯಾಗಿ ತಿಳಿದಿಲ್ಲ

1.5 ತಂಬಾಕು ಸೇವನೆಯನ್ನು ನಿಲ್ಲಿಸಿದರೆ ಅದು ವ್ಯಕ್ತಿಯ ಆರೋಗ್ಯವನ್ನು ಉತ್ತಮ ಗೊಳಿಸುತ್ತದೆಯೇ?
- Ⓐ ಹೌದು
- Ⓑ ಇಲ್ಲ
- Ⓒ ಗೊತ್ತಿಲ್ಲ

2. ಮುಂದಿನ ಪ್ರಶ್ನೆಗಳು ತಂಬಾಕು ವಿರೋಧಿ ನೀತಿಗಳ ಬಗ್ಗೆ ಆಗಿವೆ

2.1 ಸಾರ್ವಜನಿಕ ಸ್ಥಳಗಳಲ್ಲಿ ಧೂಮಪಾನ ಮಾಡುವುದನ್ನು ತಡೆಗಟ್ಟಲು ಕಾನೂನುಗಳಿವೆಯೇ?
- Ⓐ ಹೌದು
- Ⓑ ಇಲ್ಲ
- Ⓒ ಗೊತ್ತಿಲ್ಲ

2.2 ನಮ್ಮ ರಾಜ್ಯದಲ್ಲಿ ಹೊಗೆರಹಿತ ತಂಬಾಕನ್ನು(ಉದಾ: ಗುಟ್ಕ)ಜನರು ಮಾರಾಟ ಮಾಡುವುದನ್ನು ತಡೆಗಟ್ಟಲು ಕಾನೂನುಗಳಿವೆಯೇ?
- Ⓐ ಹೌದು
- Ⓑ ಇಲ್ಲ
- Ⓒ ಗೊತ್ತಿಲ್ಲ

2.3 ದೂರದರ್ಶನದ ಚಾನಲ್‌ಗಳಲ್ಲಿ ಮತ್ತು ಪತ್ರಿಕಾ ಮಾಧ್ಯಮಗಳಲ್ಲಿ ತಂಬಾಕಿನ ಬಗ್ಗೆ ಜಾಹೀರಾತುಗಳನ್ನು ತಡೆಗಟ್ಟಲು ಕಾನೂನು ಇದೆಯೇ?
- Ⓐ ಹೌದು
- Ⓑ ಇಲ್ಲ
- Ⓒ ಗೊತ್ತಿಲ್ಲ

2.4 ನಿಮ್ಮ ಶಾಲೆಯಲ್ಲಿ "ಧೂಮಪಾನರಹಿತ ಪ್ರದೇಶ – ಧೂಮಪಾನ ಮಾಡುವುದು ಅಪರಾಧ" ಎಂಬ ಫಲಕ ಇದೆಯೇ?
- Ⓐ ಹೌದು
- Ⓑ ಇಲ್ಲ
- Ⓒ ಸರಿಯಾಗಿ ಗೊತ್ತಿಲ್ಲ

2.5 ನಿಮ್ಮ ಶಾಲೆಯ ಆವರಣದ ಮುಖ್ಯದ್ವಾರದ ಬಳಿ ಪ್ರಮುಖ ಪ್ರದೇಶದಲ್ಲಿ "ತಂಬಾಕು ಮುಕ್ತ ಶಾಲೆ" ಅಥವಾ "ತಂಬಾಕು ಮುಕ್ತ ಸಂಸ್ಥೆ" ಎಂಬ ಫಲಕ ಇದೆಯೇ?
- Ⓐ ಹೌದು
- Ⓑ ಇಲ್ಲ
- Ⓒ ಸರಿಯಾಗಿ ಗೊತ್ತಿಲ್ಲ

2.6 ತಂಬಾಕಿನ ಪೊಟ್ಟಣದ ಮೇಲೆ ತಂಬಾಕಿನ ದುಷ್ಪರಿಣಾಮಗಳ ಬಗ್ಗೆ ಇರುವ ಚಿತ್ರ ಅಥವಾ ಲಿಖಿತ ಎಚ್ಚರಿಕೆಯನ್ನು ಗಮನಿಸಿದ್ದೀರಾ?
- Ⓐ ಹೌದು
- Ⓑ ಇಲ್ಲ
- Ⓒ ನಾನು ಯಾವುದೇ ತಂಬಾಕಿನ ಪೊಟ್ಟಣ ನೋಡಿಲ್ಲ

2.7 ನಿಮ್ಮ ಶಾಲೆಯ ಹತ್ತಿರ ತಂಬಾಕು ಬಳಕೆ ಮಾಡುವುದನ್ನು ತೋರಿಸುವ ಜಾಹೀರಾತು ಫಲಕವನ್ನು ಗಮನಿಸಿದ್ದೀರಾ?
- Ⓐ ಹೌದು
- Ⓑ ಇಲ್ಲ

2.8 ಕಳೆದ 30 ದಿನಗಳಲ್ಲಿ, ನಿಮ್ಮ ಶಾಲೆಯ ಸುತ್ತಮುತ್ತ ಉಚಿತವಾಗಿ ತಂಬಾಕು ಉತ್ಪನ್ನಗಳ ಹಂಚಿಕೆ ಮಾಡುವುದನ್ನು ಗಮನಿಸಿದ್ದೀರಾ?
- Ⓐ ಹೌದು
- Ⓑ ಇಲ್ಲ

2.9 ಕಳೆದ 30 ದಿನಗಳಲ್ಲಿ, ನಿಮ್ಮ ಶಾಲಾ ಆವರಣದ 100 ಯಾರ್ಡ(90 ಮೀಟರ್) ಅಂತರದಲ್ಲಿ ತಂಬಾಕು ಉತ್ಪನ್ನಗಳ ಮಾರಾಟ ಮಾಡುವುದನ್ನು ಗಮನಿಸಿದ್ದೀರಾ?
- Ⓐ ಹೌದು
- Ⓑ ಇಲ್ಲ

3. ಮುಂದಿನ ಪ್ರಶ್ನೆಗಳು ಚಲನಚಿತ್ರಗಳು ಮತ್ತು ಮ್ಯೂಸಿಕ್ ವೀಡಿಯೋಗಳ ಬಗ್ಗೆಯಾಗಿದೆ (ನಿಮಗೆ ಅನ್ವಯಿಸುವ ಎಲ್ಲಾ ವೃತ್ತಗಳಲ್ಲಿ ಗುರುತಿಸಿ)

3.1 ಈ ಕೆಳಗಿನ ಯಾವ ಕನ್ನಡ ಚಲನಚಿತ್ರಗಳನ್ನು ವೀಕ್ಷಿಸಿದ್ದೀರಿ?
- Ⓐ ಕಿರಿಕ್ ಪಾರ್ಟಿ
- Ⓑ ಮಾಸ್ಟರ್‌ಪೀಸ್
- Ⓒ ಗೋಧಿ ಬಣ್ಣ ಸಾಧಾರಣ ಮೈಕಟ್ಟು
- Ⓓ ಜಗ್ಗುದಾದ
- Ⓔ ರಂಗಿತರಂಗ
- Ⓕ ರಣವಿಕ್ರಮ
- Ⓖ ವಿರಾಟ್
- Ⓗ ರನ್ನ
- Ⓘ ಐರಾವತ
- Ⓙ ಕೋಟಿಗೊಬ್ಬ-2
- Ⓚ ಮೇಲ್ಕಂಡ ಯಾವುದೇ ಚಲನಚಿತ್ರ ನೋಡಿಲ್ಲ

3.2 ಈ ಕೆಳಗಿನ ಯಾವ ಹಿಂದಿ ಚಲನಚಿತ್ರಗಳನ್ನು ವೀಕ್ಷಿಸಿದ್ದೀರಿ?
- Ⓐ ಡಿಟೆಕ್ಟಿವ್ ಬ್ಯೊಮಕೇಶ್ ಬಕ್ಷಿ
- Ⓑ ಕಬಾಲಿ
- Ⓒ ರುಸ್ತಮ್
- Ⓓ ವೆಲ್‌ಕಮ್ ಬ್ಯಾಕ್
- Ⓔ ಶಿವಾಯ್
- Ⓕ ಬೇಬಿ
- Ⓖ ಎ ದಿಲ್ ಹೈ ಮುಶ್ಕಿಲ್
- Ⓗ ಪ್ರೇಮ್‌ರತನ್‌ಧನ್ ಪಾಯೋ
- Ⓘ ಮೇಲ್ಕಂಡ ಯಾವುದೇ ಚಲನಚಿತ್ರ ನೋಡಿಲ್ಲ

3.3 ಈ ಕೆಳಗಿನ ಯಾವ ತುಳು ಚಲನಚಿತ್ರಗಳನ್ನು ವೀಕ್ಷಿಸಿದ್ದೀರಿ?
- Ⓐ ದಬಕ್‌ದಬಾ ಐಸಾ
- Ⓑ ಚಂಡಿಕೋರಿ
- Ⓒ ಎಕ್ಕ ಸಕ
- Ⓓ ಮೇಲ್ಕಂಡ ಯಾವುದೇ ಚಲನಚಿತ್ರ ನೋಡಿಲ್ಲ

3.4 ಈ ಕೆಳಗಿನ ಯಾವ ಅನ್ಯ ಭಾಷಾ ಚಲನಚಿತ್ರಗಳನ್ನು ವೀಕ್ಷಿಸಿದ್ದೀರಿ?
- Ⓐ ಕಬಾಲಿ (ತಮಿಳು)
- Ⓑ ಐ (ತಮಿಳು)
- Ⓒ ತೆರಿ (ತಮಿಳು)
- Ⓓ ಪುಲಿಮುರುಗನ್ (ಮಲಯಾಳಂ)
- Ⓔ ಶ್ರೀಮಂತುಡು (ತೆಲುಗು)
- Ⓕ ಲೋಗನ್ (ಆಂಗ್ಲ)
- Ⓖ ಮೇಲ್ಕಂಡ ಯಾವುದೇ ಚಲನಚಿತ್ರ ನೋಡಿಲ್ಲ

3.5 ಈ ಕೆಳಗಿನ ಯಾವುದಾದರೂ ಹಿಂದಿ ಮ್ಯೂಸಿಕ್ ವೀಡಿಯೋಗಳನ್ನು ನೋಡಿದ್ದೀರಾ?
- Ⓐ ಬೊಲ್ನಾ ಮಾಹಿ ಬೊಲ್ನಾ
- Ⓑ ಧೀರೆ ಧೀರೆ ಸೆ
- Ⓒ ಹಮಾರಿ ಅಧೂರೀ ಕಹಾನಿ
- Ⓓ ಜೀನಾ ಜೀನಾ
- Ⓔ ಮೇಲ್ಕಂಡ ಯಾವುದೇ ಮ್ಯೂಸಿಕ್ ವೀಡಿಯೋಗಳನ್ನು ನೋಡಿಲ್ಲ

3.6 ಈ ಕೆಳಗಿನ ಯಾವುದಾದರೂ ಕನ್ನಡ ಮ್ಯೂಸಿಕ್ ವೀಡಿಯೋಗಳನ್ನು ನೋಡಿದ್ದೀರಾ?
- Ⓐ ನೀ ಮುದ್ದಾದ
- Ⓑ ಹುಡುಗಿಕಣ್ಣು
- Ⓒ ಮೇಲ್ಕಂಡ ಯಾವುದೇ ಮ್ಯೂಸಿಕ್ ವೀಡಿಯೋಗಳನ್ನು ನೋಡಿಲ್ಲ

3.7 ನೀವು ಚಲನಚಿತ್ರ/ಮ್ಯೂಸಿಕ್ ವೀಡಿಯೋಗಳನ್ನು ವೀಕ್ಷಿಸುವಾಗ ಯಾವುದೇ ರೀತಿಯ ತಂಬಾಕು ವಿರೋಧಿ ಸಂದೇಶಗಳನ್ನು ಗಮನಿಸಿದ್ದೀರಾ ?
- Ⓐ ಹೌದು
- Ⓑ ಇಲ್ಲ

3.8 ನೀವು ನೋಡಿದ ಚಲನಚಿತ್ರ/ಮ್ಯೂಸಿಕ್ ವೀಡಿಯೋಗಳಲ್ಲಿ ನಟ/ನಟಿಯರು ಧೂಮಪಾನ ಮಾಡುವುದನ್ನು ಗಮನಿಸಿದ್ದೀರಾ ?
- Ⓐ ಹೌದು
- Ⓑ ಇಲ್ಲ

3.9 ನೀವು ನೋಡಿದ ಚಲನಚಿತ್ರ/ಮ್ಯೂಸಿಕ್ ವೀಡಿಯೋಗಳಲ್ಲಿ ನಟ/ನಟಿಯರು ಹೊಗೆರಹಿತ ತಂಬಾಕಿನ ಬಳಕೆ ಮಾಡುವುದನ್ನು ಗಮನಿಸಿದ್ದೀರಾ ?
- Ⓐ ಹೌದು
- Ⓑ ಇಲ್ಲ

4. ಈ ಮುಂದಿನ ಪ್ರಶ್ನೆಗಳು ಅಂಗಡಿಗಳ ಕುರಿತಾಗಿದೆ

4.1 ನೀವು ಸೂಪರ್ ಮಾರ್ಕೆಟ್‌ಗಳಿಗೆ ಹೋದಾಗ ತಂಬಾಕು ಉತ್ಪನ್ನಗಳನ್ನು ಮಾರಾಟ ಮಾಡಲು ಇಟ್ಟಿರುವುದನ್ನು ಎಷ್ಟು ಸಲ ಗಮನಿಸಿದ್ದೀರಿ ?
- Ⓐ ಪ್ರತಿಸಲ
- Ⓑ ಹೆಚ್ಚಿನ ಸಲ
- Ⓒ ಕೆಲವು ಸಲ
- Ⓓ ಯಾವಾಗಲಾದರೊಮ್ಮೆ
- Ⓔ ಯಾವತ್ತೂ ಇಲ್ಲ
- Ⓕ ನಾನು ಸೂಪರ್ ಮಾರ್ಕೆಟ್‌ಗಳಿಗೆ ಹೋಗುವುದಿಲ್ಲ

4.2 ನೀವು ಸಣ್ಣ ಅಂಗಡಿಗಳಿಗೆ(ಸಣ್ಣ ದಿನಸಿಅಂಗಡಿಗಳೂ, ಪಾನ್‌ಶಾಪ್‌ಗಳು) ಹೋದಾಗ ಎಷ್ಟು ಸಲ ತಂಬಾಕು ಉತ್ಪನ್ನಗಳನ್ನು ಮಾರಲು ಇಟ್ಟಿರುವುದನ್ನು ಗಮನಿಸಿದ್ದೀರಿ ?
- Ⓐ ಪ್ರತಿಸಲ
- Ⓑ ಹೆಚ್ಚಿನ ಸಲ
- Ⓒ ಕೆಲವು ಸಲ
- Ⓓ ಯಾವಾಗಲಾದರೊಮ್ಮೆ
- Ⓔ ಯಾವತ್ತೂ ಇಲ್ಲ
- Ⓕ ನಾನು ಸಣ್ಣಅಂಗಡಿಗಳಿಗೆ ಹೋಗುವುದಿಲ್ಲ

4.3 ನೀವು ಸೂಪರ್ ಮಾರ್ಕೆಟ್ ಅಥವಾ ಸಣ್ಣ ಅಂಗಡಿಗಳಿಗೆ ಹೋದಾಗ ಯಾವುದೇ ರೀತಿಯ ತಂಬಾಕಿನ ಬ್ರಾಂಡ್‌ಗಳನ್ನು ಪ್ರದರ್ಶನಕ್ಕೆ ಇಟ್ಟಿರುವುದನ್ನು ಗಮನಿಸಿದ್ದೀರಾ?
- Ⓐ ಹೌದು, ನಾನು ಗಮನಿಸಿದ್ದೇನೆ
  ನಿಮ್ಮಉತ್ತರ ಹೌದಾದಲ್ಲಿ, ಬ್ರಾಂಡ್‌ನ್ನು ತಿಳಿಸಿ________________________
  ____
- Ⓑ ಇಲ್ಲ, ನಾನು ಯಾವುದೇ ಬ್ರಾಂಡ್‌ಗಳನ್ನು ಪ್ರದರ್ಶನಕ್ಕೆ ಇಟ್ಟಿರುವುದನ್ನು ಯಾವತ್ತು ನೋಡಿಲ್ಲ
- Ⓒ ನನಗೆ ಯಾವುದೇ ಬ್ರಾಂಡ್‌ಗಳು ನೆನೆಪಿನಲ್ಲಿ ಇಲ್ಲ
- Ⓓ ನಾನು ಅಂಗಡಿ/ಸೂಪರ್ ಮಾರ್ಕೆಟ್‌ಗಳಿಗೆ ಹೋಗುವುದಿಲ್ಲ

4.4 ಒಂದು ವೇಳೆ ನಿಮ್ಮ ಪ್ರಾಯದವರು ಅಂಗಡಿಗಳಲ್ಲಿ ತಂಬಾಕು ಉತ್ಪನ್ನಗಳನ್ನು ಖರೀದಿಸಲು ಪ್ರಯತ್ನಿಸಿದಲ್ಲಿ, ನಿಮ್ಮ ಪ್ರಕಾರ ಅವರು ಸಫಲರಾಗುತ್ತಾರೆಯೇ ?
- Ⓐ ಹೌದು
- Ⓑ ಇಲ್ಲ
- Ⓒ ಗೊತ್ತಿಲ್ಲ

5. ಮುಂದಿನ ಪ್ರಶ್ನೆಗಳು ಧೂಮಪಾನದ ಬಗ್ಗೆಯಾಗಿದೆ

5.1 ನಿಮ್ಮ ಮನೆಯಲ್ಲಿ ಧೂಮಪಾನ ಬಳಕೆಗೆ ಅವಕಾಶವಿದೆಯೇ?
- Ⓐ ಹೌದು
- Ⓑ ಇಲ್ಲ

5.2 ನಿಮ್ಮ ಕುಟುಂಬದಲ್ಲಿ ಯಾರಾದರೂ ಧೂಮಪಾನ ಬಳಸುತ್ತಾರೆಯೇ (ನಿಮಗೆ ಅನ್ವಯಿಸುವ ಎಲ್ಲಾ ವೃತ್ತಗಳಲ್ಲಿ ಗುರುತಿಸಿ)
- Ⓐ ಯಾರೂಇಲ್ಲ
- Ⓑ ತಾಯಿ
- Ⓒ ತಂದೆ
- Ⓓ ಸಹೋದರ
- Ⓔ ಸಹೋದರಿ
- Ⓕ ಇತರ

5.3 ನಿಮ್ಮ ಎಷ್ಟು ಸ್ನೇಹಿತರು ಧೂಮಪಾನ ಮಾಡುತ್ತಾರೆ?
- Ⓐ ಯಾರೂಇಲ್ಲ
- Ⓑ ಒಬ್ಬ
- Ⓒ ಇಬ್ಬರೂ
- Ⓓ ಮೂವರು ಅಥವಾ ಹೆಚ್ಚು
- Ⓔ ಸರಿಯಾಗಿ ಗೊತ್ತಿಲ್ಲ

5.4 ಶಾಲಾ ಕಟ್ಟಡ ಅಥವಾ ಶಾಲಾ ಆವರಣದಲ್ಲಿ ಯಾರಾದರೂ ಧೂಮಪಾನ ಮಾಡುವುದನ್ನು ನೋಡಿದ್ದೀರಾ?
- Ⓐ ಹೌದು
- Ⓑ ಇಲ್ಲ

5.5 ಈ ಕೆಳಗಿನ ಹೇಳಿಕೆಗಳನ್ನು ಗಮನವಿಟ್ಟು ಓದಿ ಮತ್ತು ನಿಮ್ಮ ಕುರಿತು ಧೂಮಪಾನದ ಬಗ್ಗೆ ಅನ್ವಯವಾಗುವ ಆಯ್ಕೆಯನ್ನು ಗುರುತಿಸಿ.
- Ⓐ ನಾನು ಯಾವತ್ತು ಧೂಮಪಾನ ಮಾಡಲಿಲ್ಲ
- Ⓑ ನಾನು ಹಿಂದೆ ಧೂಮಪಾನ ಮಾಡಿದ್ದೆ, ಆದರೆ ಕಳೆದ 30 ದಿನಗಳಲ್ಲಿ ಮಾಡಿಲ್ಲ
- Ⓒ ನಾನು ಕೆಲವೊಮ್ಮೆ ಧೂಮಪಾನ ಮಾಡುತ್ತೇನೆ, ಆದರೆ ವಾರಕ್ಕೆ 1 ಬಾರಿಗಿಂತ ಕಡಿಮೆ
- Ⓓ ನಾನು ವಾರದಲ್ಲಿ 1 ರಿಂದ 6 ಬಾರಿ ಧೂಮಪಾನ ಮಾಡುತ್ತೇನೆ
- Ⓔ ನಾನು ವಾರದಲ್ಲಿ 6ಕ್ಕಿಂತ ಹೆಚ್ಚಿನ ಬಾರಿ ಧೂಮಪಾನ ಮಾಡುತ್ತೇನೆ

5.6 ಪ್ರಥಮ ಬಾರಿಗೆ ಧೂಮಪಾನ ಪ್ರಯತ್ನಿಸಿದಾಗ ನಿಮ್ಮ ಪ್ರಾಯ?
- Ⓐ ನಾನು ಧೂಮಪಾನ ಮಾಡಲು ಪ್ರಯತ್ನಿಸಿಲ್ಲ
- Ⓑ 7 ವರ್ಷ ಅಥವಾ ಕಡಿಮೆ ಪ್ರಾಯ
- Ⓒ 8 ವರ್ಷ ಪ್ರಾಯ
- Ⓓ 9 ವರ್ಷ ಪ್ರಾಯ
- Ⓔ 10 ವರ್ಷ ಪ್ರಾಯ
- Ⓕ 11 ವರ್ಷ ಪ್ರಾಯ
- Ⓖ 12 ವರ್ಷ ಪ್ರಾಯ
- Ⓗ 13 ವರ್ಷ ಪ್ರಾಯ
- Ⓘ 14 ವರ್ಷ ಪ್ರಾಯ
- Ⓙ 15 ವರ್ಷ ಅಥವಾ ಹೆಚ್ಚಿನ ಪ್ರಾಯ

5.7 ನೀವು ಮೊದಲ ಬಾರಿ ಯಾವ ವಿಧದ ಧೂಮಪಾನ ಮಾಡಿದ್ದೀರಿ?
- Ⓐ ನಾನು ಯಾವತ್ತಿಗೂ ಧೂಮಪಾನ ಮಾಡಿಲ್ಲ
- Ⓑ ನಾನು ಸಿಗರೇಟ್ ಸೇದಿದ್ದೇನೆ
- Ⓒ ನಾನು ಬೀಡಿ ಸೇದಿದ್ದೇನೆ
- Ⓓ ನಾನು ಹುಕ್ಕಾ ಸೇದಿದ್ದೇನೆ
- Ⓔ ಬೇರೆ ಯಾವುದಾದರೆ, ತಿಳಿಸಿ____________________________

5.8 ನೀವು ಧೂಮಪಾನ ಆರಂಭಿಸಲು ಮುಖ್ಯ ಕಾರಣವೇನು?
- Ⓐ ನಾನು ಯಾವತ್ತಿಗೂ ಧೂಮಪಾನ ಮಾಡಿಲ್ಲ
- Ⓑ ಒಂಟಿತನ
- Ⓒ ಸ್ನೇಹಿತರ ಒತ್ತಾಯ
- Ⓓ ಹಿರಿಯರಿಂದ ಕಲಿತೆ
- Ⓔ ಕುತೂಹಲ
- Ⓕ ಪ್ರೌಢರಾಗಿ ಕಾಣಲು
- Ⓖ ಒತ್ತಡ
- Ⓗ ಹೆಚ್ಚು ಸ್ನೇಹಿತರನ್ನು ಹೊಂದಲು
- Ⓘ ಆಕರ್ಷಕವಾಗಿ ಕಾಣಲು
- Ⓙ ನಟ/ನಟಿಯರ ಧೂಮಪಾನ ಮಾಡುವುದು
- Ⓚ ಇತರೆ ಕಾರಣಗಳಿದ್ದರೆ, ತಿಳಿಸಿ: ____________________________

5.9 ನೀವು ಮೊದಲ ಬಾರಿ ಸಿಗರೇಟ್/ಬೀಡಿಗಳನ್ನು ಯಾವ ರೀತಿಯಲ್ಲಿ ಪಡೆದುಕೊಂಡಿರಿ?

- Ⓐ ನಾನು ಧೂಮಪಾನದ ಬಳಕೆ ಮಾಡಿಲ್ಲ
- Ⓑ ನಾನು ಅಂಗಡಿಯಲ್ಲಿ ಖರೀದಿಸಿದೆ
- Ⓒ ನಾನು ಇಂಟರ್ನೆಟ್/ಆನ್‌ಲೈನ್ ಮೂಲಕ ಪಡೆದುಕೊಂಡೆ
- Ⓓ ನಾನು ಬೇರೆಯವರಿಗೆ ಹಣಕೊಟ್ಟು ನನಗೋಸ್ಕರ ಖರೀದಿಸಿದೆ
- Ⓔ ನಾನು ಇನ್ನೊಬ್ಬರಿಂದ ಕೇಳಿ ಪಡೆದುಕೊಂಡೆ
- Ⓕ ಕುಟುಂಬದ ಸದಸ್ಯರಿಂದ ಪಡೆದುಕೊಂಡೆ
- Ⓖ ನಾನು ಭಾಗಶ: ಉಳಿದ ಸಿಗರೇಟ್/ಬೀಡಿಯನ್ನು ಸೇದಿದೆ
- Ⓗ ಬೇರೆ ಇದ್ದಲ್ಲಿ, ತಿಳಿಸಿ________________________________

6. ಮುಂದಿನ ಪ್ರಶ್ನೆಗಳು ಒಂದು ತಿಂಗಳ (30 ದಿನಗಳ) ಹಿಂದಿನ ಧೂಮಪಾನದ ಬಗ್ಗೆಯಾಗಿದೆ

6.1 ನೀವು ಕಳೆದ 30 ದಿನಗಳಲ್ಲಿ ಎಷ್ಟು ಬಾರಿ ಧೂಮಪಾನ ಮಾಡಿದ್ದೀರಿ?

- Ⓐ ನಾನು ಧೂಮಪಾನದ ಬಳಕೆ ಮಾಡಿಲ್ಲ
- Ⓑ ವಾರದಲ್ಲಿ ಒಂದು ಸಲಕ್ಕಿಂತ ಕಡಿಮೆ
- Ⓒ ವಾರದಲ್ಲಿ ಒಂದರಿಂದ ಮೂರು ಸಲ
- Ⓓ ವಾರದಲ್ಲಿ ನಾಲ್ಕರಿಂದ ಆರು ಸಲ
- Ⓔ ವಾರದಲ್ಲಿ ಆರು ಸಲಕ್ಕಿಂತ ಜಾಸ್ತಿ

6.2 ನೀವು ಕಳೆದ 30 ದಿನಗಳಲ್ಲಿ (ಒಂದು ತಿಂಗಳು) ನಿಮಗೋಸ್ಕರ ಎಷ್ಟು ಸಿಗರೇಟ್‌ಗಳನ್ನು ಖರೀದಿಸಿದ್ದೀರಿ?

- Ⓐ ಒಂದೂ ಇಲ್ಲ
- Ⓑ ಒಂದು ಸಿಗರೇಟ್
- Ⓒ 2-9 ಸಿಗರೇಟ್
- Ⓓ 10 ಸಿಗರೇಟ್‌ಗಳ ಒಂದು ಪ್ಯಾಕ್
- Ⓔ 20 ಸಿಗರೇಟ್‌ಗಳ ಒಂದು ಪ್ಯಾಕ್
- Ⓕ 20ಕ್ಕಿಂತ ಹೆಚ್ಚು ಸಿಗರೇಟ್‌ಗಳು

6.3 ಕಳೆದ 30 ದಿನಗಳಲ್ಲಿ ನಿಮಗೋಸ್ಕರ ಸೇದುವ ಸಿಗರೇಟಿಗೆ ಎಷ್ಟು ಹಣ ಖರ್ಚು ಮಾಡಿದ್ದೀರಿ?

- Ⓐ ಏನೂ ಖರ್ಚು ಮಾಡಿಲ್ಲ
- Ⓑ ನಾನು ಸೇದುತ್ತೇನೆ ಆದರೆ ಖರೀದಿಸುವುದಿಲ್ಲ
- Ⓒ 10 ರೂ. ಗಿಂತ ಕಡಿಮೆ
- Ⓓ ರೂ. 11 ರಿಂದ 30
- Ⓔ ರೂ. 31 ರಿಂದ 60
- Ⓕ ರೂ. 61 ರಿಂದ 100
- Ⓖ ರೂ. 100 ಕ್ಕಿಂತ ಹೆಚ್ಚು

6.4 ನೀವು ಕಳೆದ 30 ದಿನಗಳಲ್ಲಿ (ಒಂದು ತಿಂಗಳು) ನಿಮಗೋಸ್ಕರ ಎಷ್ಟು ಬೀಡಿಗಳನ್ನು ಖರೀದಿಸಿದ್ದೀರಿ?

- Ⓐ ಒಂದೂ ಇಲ್ಲ
- Ⓑ ಒಂದು ಬೀಡಿ
- Ⓒ 2-5 ಬೀಡಿಗಳು
- Ⓓ 6-10 ಬೀಡಿಗಳು
- Ⓔ 11-20 ಬೀಡಿಗಳು
- Ⓕ 25 ಬೀಡಿಗಳ ಒಂದು ಪ್ಯಾಕ್

6.5 ಕಳೆದ 30 ದಿನಗಳಲ್ಲಿ ನಿಮಗೋಸ್ಕರ ಸೇದುವ ಬೀಡಿಗಳಿಗೆ ಎಷ್ಟು ಹಣ ಖರ್ಚು ಮಾಡಿದ್ದೀರಿ?

- Ⓐ ಏನೂ ಖರ್ಚು ಮಾಡಿಲ್ಲ
- Ⓑ ನಾನು ಸೇದುತ್ತೇನೆ ಆದರೆ ಖರೀದಿಸಿಲ್ಲ
- Ⓒ 10 ರೂ. ಗಿಂತ ಕಡಿಮೆ
- Ⓓ ರೂ. 11 ರಿಂದ 20
- Ⓔ ರೂ. 21 ರಿಂದ 30
- Ⓕ ರೂ. 30 ಕ್ಕಿಂತ ಹೆಚ್ಚು

6.6 ನೀವು ಕಳೆದ 07 ದಿನಗಳಲ್ಲಿ ಎಷ್ಟು ಸಿಗರೇಟ್‌ಗಳನ್ನು ಸೇದಿದ್ದೀರಿ?

- Ⓐ ಸೇದಲೇ ಇಲ್ಲ
- Ⓑ 1-2
- Ⓒ 3-5
- Ⓓ 6 ಕ್ಕಿಂತ ಜಾಸ್ತಿ

6.7 ನೀವು ಕಳೆದ 07 ದಿನಗಳಲ್ಲಿ ಎಷ್ಟು ಬೀಡಿಗಳನ್ನು ಸೇದಿದ್ದೀರಿ?

- Ⓐ ಸೇದಲೇ ಇಲ್ಲ
- Ⓑ 1-2
- Ⓒ 3-5
- Ⓓ 6 ಕ್ಕಿಂತ ಜಾಸ್ತಿ

7. ಮುಂದೆ ಬರುವ ಪ್ರಶ್ನೆಗಳು ಧೂಮಪಾನದ ಬಳಕೆಯ ಬಗ್ಗೆ ನಿಮ್ಮ ಯೋಜನೆಗಳು ಮತ್ತು ಆಲೋಚನೆಗಳ ಬಗ್ಗೆ ಆಗಿವೆ

7.1 ಶೀಘ್ರದಲ್ಲಿ ನೀವು ಧೂಮಪಾನ ಮಾಡಲು ಆರಂಭಿಸುತ್ತೀರಿ ಎಂದು ನಿಮಗೆ ಅನಿಸುತ್ತದೆಯೇ?

- Ⓐ ಹೌದು
- Ⓑ ಇಲ್ಲ

7.2 ನಿಮ್ಮ ಆತ್ಮೀಯ ಸ್ನೇಹಿತರಲ್ಲಿ ಒಬ್ಬ ನಿಮಗೆ ಸಿಗರೇಟ್ ನೀಡಿದಲ್ಲಿ ನೀವು ಅದನ್ನು ಬಳಸುತ್ತೀರಾ?

- Ⓐ ಖಂಡಿತವಾಗಿಯೂ ಹೌದು
- Ⓑ ಪ್ರಾಯಶ: ಹೌದು
- Ⓒ ಪ್ರಾಯಶ: ಇಲ್ಲ
- Ⓓ ಖಂಡಿತವಾಗಿಯೂ ಇಲ್ಲ

7.3 ನೀವು ಮುಂದಿನ ವರ್ಷ ಯಾವತ್ತಾದರೂ ಧೂಮಪಾನ ಮಾಡಬಹುದು ಎಂದು ಭಾವಿಸುತ್ತೀರಾ?

- Ⓐ ಖಂಡಿತವಾಗಿಯೂ ಹೌದು
- Ⓑ ಪ್ರಾಯಶ: ಹೌದು
- Ⓒ ಪ್ರಾಯಶ: ಇಲ್ಲ
- Ⓓ ಖಂಡಿತವಾಗಿಯೂ ಇಲ್ಲ

7.4 ಮುಂದೆ ನೀವು ಕಾಲೇಜು ಹೋಗಲು ಪ್ರಾರಂಭಿಸಿದಾಗ ಧೂಮಪಾನ ಮಾಡುವ ಆಲೋಚನೆ ಇದೆಯೇ?

- Ⓐ ಖಂಡಿತವಾಗಿಯೂ ಹೌದು
- Ⓑ ಪ್ರಾಯಶ: ಹೌದು
- Ⓒ ಪ್ರಾಯಶ: ಇಲ್ಲ
- Ⓓ ಖಂಡಿತವಾಗಿಯೂ ಇಲ್ಲ

8. ಮುಂದಿನ ಪ್ರಶ್ನೆಗಳು ಹೊಗೆರಹಿತ ತಂಬಾಕು ಬಳಕೆಯ ಬಗ್ಗೆಯಾಗಿದೆ

(ಜಗಿಯುವ ತಂಬಾಕು, ಗುಟ್ಕಾ ಖೈನಿ, ಜರ್ದಾ, ನಶ್ಯ)

8.1 ನಿಮ್ಮ ಮನೆಯಲ್ಲಿ ಹೊಗೆರಹಿತ ತಂಬಾಕಿನ ಬಳಕೆಗೆ ಅವಕಾಶವಿದೆಯೇ ?

- Ⓐ ಹೌದು
- Ⓑ ಇಲ್ಲ

8.2 ನಿಮ್ಮ ಕುಟುಂಬದಲ್ಲಿ ಯಾರಾದರೂ ಹೊಗೆರಹಿತ ತಂಬಾಕು ಬಳಸುತ್ತಾರೆಯೇ? (ನಿಮಗೆ ಅನ್ವಯಿಸುವ ಎಲ್ಲಾ ವೃತ್ತಗಳಲ್ಲಿ ಗುರುತಿಸಿ)

- Ⓐ ಯಾರೂಇಲ್ಲ
- Ⓑ ತಾಯಿ
- Ⓒ ತಂದೆ
- Ⓓ ಸಹೋದರ
- Ⓔ ಸಹೋದರಿ
- Ⓕ ಇತರ

8.3 ನಿಮ್ಮ ಎಷ್ಟು ಸ್ನೇಹಿತರು ಹೊಗೆರಹಿತ ತಂಬಾಕು ಬಳಸುತ್ತಾರೆ?

- Ⓐ ಯಾರೂಇಲ್ಲ
- Ⓑ ಒಬ್ಬ
- Ⓒ ಇಬ್ಬರೂ
- Ⓓ ಮೂವರು ಅಥವಾ ಹೆಚ್ಚು
- Ⓔ ಸರಿಯಾಗಿ ಗೊತ್ತಿಲ್ಲ

8.4 ಈ ಕೆಳಗಿನ ಹೇಳಿಕೆಗಳನ್ನು ಗಮನವಿಟ್ಟು ಓದಿ ಮತ್ತು ನಿಮ್ಮ ಕುರಿತು ಹೊಗೆರಹಿತ ತಂಬಾಕಿನ ಬಗ್ಗೆ ಅನ್ವಯವಾಗುವ ಆಯ್ಕೆಯನ್ನು ಗುರುತಿಸಿ.

- Ⓐ ನಾನು ಯಾವತ್ತು ಹೊಗೆರಹಿತ ತಂಬಾಕಿನ ಬಳಕೆ ಮಾಡಲಿಲ್ಲ
- Ⓑ ನಾನು ಹಿಂದೆ ಹೊಗೆರಹಿತ ತಂಬಾಕು ಬಳಸಿದ್ದೇನೆ, ಆದರೆ ಕಳೆದ 30ದಿನಗಳಲ್ಲಿ ಇಲ್ಲ
- Ⓒ ನಾನು ಕೆಲವೊಮ್ಮೆ ಹೊಗೆರಹಿತ ತಂಬಾಕು ಬಳಸುತ್ತೇನೆ, ಆದರೆ ವಾರಕ್ಕೆ 1 ಬಾರಿಗಿಂತ ಕಡಿಮೆ
- Ⓓ ನಾನು ವಾರದಲ್ಲಿ 1 ರಿಂದ 6 ಬಾರಿ ಹೊಗೆರಹಿತ ತಂಬಾಕು ಬಳಸುತ್ತೇನೆ.
- Ⓔ ನಾನು ವಾರದಲ್ಲಿ 6 ಕ್ಕಿಂತ ಹೆಚ್ಚಿನ ಬಾರಿ ಹೊಗೆರಹಿತ ತಂಬಾಕು ಬಳಸುತ್ತೇನೆ.

8.5 ಪ್ರಥಮ ಬಾರಿ ಹೊಗೆರಹಿತ ತಂಬಾಕಿನ ಬಳಕೆ ಪ್ರಯತ್ನಿಸಿದಾಗ ನಿಮ್ಮ ಪ್ರಾಯ?

- Ⓐ ನಾನು ಪ್ರಯತ್ನಿಸಿಲ್ಲ
- Ⓑ 7 ವರ್ಷ ಅಥವಾ ಕಡಿಮೆ ಪ್ರಾಯ
- Ⓒ 8 ವರ್ಷ ಪ್ರಾಯ
- Ⓓ 9 ವರ್ಷ ಪ್ರಾಯ
- Ⓔ 10 ವರ್ಷ ಪ್ರಾಯ
- Ⓕ 11 ವರ್ಷ ಪ್ರಾಯ
- Ⓖ 12 ವರ್ಷ ಪ್ರಾಯ
- Ⓗ 13 ವರ್ಷ ಪ್ರಾಯ
- Ⓘ 14 ವರ್ಷ ಪ್ರಾಯ
- Ⓙ 15 ವರ್ಷ ಅಥವಾ ಹೆಚ್ಚಿನ ಪ್ರಾಯ

8.6 ನೀವು ಮೊದಲ ಬಾರಿ ಹೊಗೆರಹಿತ ತಂಬಾಕಿನ ಬಳಕೆ ಆರಂಭಿಸಲು ಮುಖ್ಯ ಕಾರಣವೇನು?

- Ⓐ ನಾನು ಬಳಕೆ ಮಾಡಿಲ್ಲ
- Ⓑ ಒಂಟಿತನ
- Ⓒ ಸ್ನೇಹಿತರ ಒತ್ತಾಯ
- Ⓓ ಹಿರಿಯರಿಂದ ಕಲಿತೆ
- Ⓔ ಕುತೂಹಲ
- Ⓕ ಪ್ರೌಢರಾಗಿ ಕಾಣಲು
- Ⓖ ಒತ್ತಡ
- Ⓗ ಹೆಚ್ಚು ಸ್ನೇಹಿತರನ್ನು ಹೊಂದಲು
- Ⓘ ರುಚಿಯನ್ನು ಆಸ್ವಾಧಿಸಲು
- Ⓙ ಆಕರ್ಷಿತರಾಗಿ ಕಾಣಲು
- Ⓚ ಇತರೆ ಕಾರಣ ಇದ್ದಲ್ಲಿ ತಿಳಿಸಿ:____________________________

8.7 ನೀವು ಕಳೆದ 30 ದಿನಗಳಲ್ಲಿ, ಹೊಗೆರಹಿತ ತಂಬಾಕಿನ ಬಳಕೆ ಮಾಡಿದ್ದಲ್ಲಿ, ಈ ಕೆಳಗಿನವುಗಳಲ್ಲಿ ಯಾವುದನ್ನು ಬಳಸಿದ್ದೀರಿ? (ನಿಮಗೆ ಅನ್ವಯಿಸುವ ಎಲ್ಲಾ ವೃತ್ತಗಳಲ್ಲಿ ಗುರುತಿಸಿ)

- Ⓐ ನಾನು ಬಳಕೆ ಮಾಡಿಲ್ಲ
- Ⓑ ನಶ್ಯ
- Ⓒ ಖೈನಿ
- Ⓓ ಜಗಿಯುವ ತಂಬಾಕು
- Ⓔ ಗುಟ್ಕಾ
- Ⓕ ಜರ್ದಾ
- Ⓖ ಇತರೆ, ಇದ್ದಲ್ಲಿ ತಿಳಿಸಿ:________________________________

8.8 ನೀವು ಕಳೆದ 30 ದಿನಗಳಲ್ಲಿ (ಒಂದು ತಿಂಗಳು), ನಿಮಗೋಸ್ಕರ ಎಷ್ಟು ಹೊಗೆರಹಿತ ತಂಬಾಕನ್ನು ಖರೀದಿಸಿದ್ದೀರಿ?

- Ⓐ ಇಲ್ಲ, ನಾನು ಬಳಕೆ ಮಾಡಿಲ್ಲ
- Ⓑ ನಾನು ಬಳಸುತ್ತೇನೆ ಆದರೆ ಖರೀದಿಸಿಲ್ಲ
- Ⓒ ಒಂದು ಪ್ಯಾಕೆಟ್
- Ⓓ 2-5 ಪ್ಯಾಕೆಟ್
- Ⓔ 5-10 ಪ್ಯಾಕೆಟ್
- Ⓕ 10ಕ್ಕಿಂತ ಹೆಚ್ಚು ಪ್ಯಾಕೆಟ್‌ಗಳು

8.9 ನೀವು ಕಳೆದ 7 ದಿನಗಳಲ್ಲಿ ಎಷ್ಟು ಪ್ಯಾಕೆಟ್ ಹೊಗೆರಹಿತ ತಂಬಾಕನ್ನು ಜಗಿದಿದ್ದೀರಿ?

- Ⓐ ಇಲ್ಲ ನಾನು ಬಳಕೆ ಮಾಡಿಲ್ಲ
- Ⓑ ಒಂದು ಪ್ಯಾಕೆಟ್
- Ⓒ 2-5 ಪ್ಯಾಕೆಟ್
- Ⓓ 6-10 ಪ್ಯಾಕೆಟ್‌ಗಳು
- Ⓔ 10ಕ್ಕಿಂತ ಹೆಚ್ಚು ಪ್ಯಾಕೆಟ್‌ಗಳು

9. ಮುಂದಿನ ಪ್ರಶ್ನೆಗಳು ಹೊಗೆರಹಿತ ತಂಬಾಕಿನ ಬಳಕೆಯ ಬಗ್ಗೆ ನಿಮ್ಮ ಯೋಜನೆಗಳು ಮತ್ತು ಆಲೋಚನೆಗಳ ಬಗ್ಗೆ ಆಗಿವೆ

9.1 ಶೀಘ್ರದಲ್ಲಿ ನೀವು ಹೊಗೆರಹಿತ ತಂಬಾಕು ಬಳಸಲು ಆರಂಭಿಸುತ್ತೀರಿ ಎಂದು ನಿಮಗೆ ಅನಿಸುತ್ತದೆಯೇ?

- Ⓐ ಹೌದು
- Ⓑ ಇಲ್ಲ

9.2 ನಿಮ್ಮ ಆತ್ಮೀಯ ಸ್ನೇಹಿತರಲ್ಲಿ ಒಬ್ಬ ನಿಮಗೆ ಹೊಗೆರಹಿತ ತಂಬಾಕು ಕೊಟ್ಟಲ್ಲಿ ನೀವು ಬಳಸುತ್ತೀರಾ?

- Ⓐ ಖಂಡಿತವಾಗಿಯೂ ಹೌದು
- Ⓑ ಪ್ರಾಯಶ: ಹೌದು
- Ⓒ ಪ್ರಾಯಶ: ಇಲ್ಲ
- Ⓓ ಖಂಡಿತವಾಗಿಯೂ ಇಲ್ಲ

9.3 ನೀವು ಮುಂದಿನ ವರ್ಷ ಯಾವತ್ತಾದರೂ ಹೊಗೆರಹಿತ ತಂಬಾಕು ಬಳಸಬಹುದೆಂದು ಭಾವಿಸುತ್ತೀರಾ?

Ⓐ ಖಂಡಿತವಾಗಿಯೂ ಹೌದು
Ⓑ ಪ್ರಾಯಶಃ ಹೌದು
Ⓒ ಪ್ರಾಯಶಃ ಇಲ್ಲ
Ⓓ ಖಂಡಿತವಾಗಿಯೂ ಇಲ್ಲ

9.4 ಮುಂದೆ ನೀವು ಕಾಲೇಜು ಹೋಗಲು ಪ್ರಾರಂಭಿಸಿದಾಗ ಹೊಗೆರಹಿತ ತಂಬಾಕಿನ ಬಳಕೆ ಮಾಡುವ ಆಲೋಚನೆ ಇದೆಯೇ?

Ⓐ ಖಂಡಿತವಾಗಿಯೂ ಹೌದು
Ⓑ ಪ್ರಾಯಶಃ ಹೌದು
Ⓒ ಪ್ರಾಯಶಃ ಇಲ್ಲ
Ⓓ ಖಂಡಿತವಾಗಿಯೂ ಇಲ್ಲ

10. ಮುಂದಿನ ಪ್ರಶ್ನೆಗಳು ತಂಬಾಕಿನ ಬಳಕೆಯ ವರ್ಜನೆ(ನಿಲ್ಲಿಸುವುದರ) ಬಗ್ಗೆ ಆಗಿವೆ

10.1 ನೀವು ಯಾವತ್ತಾದರೂ ತಂಬಾಕಿನ ಬಳಕೆಯನ್ನು ನಿಲ್ಲಿಸುವುದಾಗಿ ಯೋಚಿಸಿದ್ದೀರಾ?

Ⓐ ಹೌದು
Ⓑ ಇಲ್ಲ
Ⓒ ನಾನು ತಂಬಾಕಿನ ಬಳಕೆ ಮಾಡಿಲ್ಲ

10.2 ನೀವು ತಂಬಾಕಿನ ಬಳಕೆ ವರ್ಜಿಸಲು ಪ್ರಯತ್ನಿಸುತ್ತಿದ್ದೀರಾ?

Ⓐ ಹೌದು
Ⓑ ಇಲ್ಲ
Ⓒ ನಾನು ತಂಬಾಕಿನ ಬಳಕೆ ಮಾಡಿಲ್ಲ

11. ಮುಂದಿನ ಪ್ರಶ್ನೆಗಳು ತಂಬಾಕು ವಿರೋಧ ಚಟುವಟಿಕೆಗಳ ಅರಿವಿನ ಬಗ್ಗೆ ಆಗಿರುತ್ತದೆ

11.1 ಕಳೆದ ಒಂದು ವರ್ಷದಲ್ಲಿ ನಿಮ್ಮ ಶಾಲೆಯಲ್ಲಿ ತಂಬಾಕು ಮತ್ತು ತಂಬಾಕು ಉತ್ಪನ್ನಗಳ ದುಷ್ಪರಿಣಾಮಗಳ ಬಗ್ಗೆ ಯಾವುದೇ ಶೈಕ್ಷಣಿಕ ತರಗತಿಗಳನ್ನು ಏರ್ಪಡಿಸಲಾಗಿತ್ತೇ?

Ⓐ ಹೌದು
Ⓑ ಇಲ್ಲ
Ⓒ ಸರಿಯಾಗಿ ತಿಳಿದಿಲ್ಲ

11.2 ನೀವು ಕೊನೆಯ ಒಂದು ವರ್ಷದಲ್ಲಿ ತಂಬಾಕು ವಿರೋಧಿ ಚಟುವಟಿಕೆಗಳಲ್ಲಿ ಭಾಗವಹಿಸಿದ್ದೀರಾ?

Ⓐ ಹೌದು
Ⓑ ಇಲ್ಲ

11.3 ನೀವು ಕಳೆದ 30 ದಿನಗಳಲ್ಲಿ ರೇಡಿಯೋ/ದೂರದರ್ಶನದಲ್ಲಿ ಎಷ್ಟು ತಂಬಾಕು ವಿರೋಧಿ ಸಂದೇಶಗಳನ್ನು ಕೇಳಿದ್ದೀರಿ/ನೋಡಿದ್ದೀರಿ?

Ⓐ ಒಂದೂ ಇಲ್ಲ
Ⓑ 1 ರಿಂದ 5 ಸಂದೇಶಗಳು
Ⓒ 6 ರಿಂದ 10 ಸಂದೇಶಗಳು
Ⓓ 10 ಕ್ಕಿಂತ ಜಾಸ್ತಿ ಸಂದೇಶಗಳು

11.4 ನೀವು ಕಳೆದ 30 ದಿನಗಳಲ್ಲಿ ಪೋಸ್ಟರ್‌ಗಳ ಮೇಲೆ ಎಷ್ಟು ತಂಬಾಕು ವಿರೋಧಿ ಸಂದೇಶಗಳನ್ನು ನೋಡಿದ್ದೀರಿ?

Ⓐ ಒಂದೂ ಇಲ್ಲ
Ⓑ 1 ರಿಂದ 5 ಸಂದೇಶಗಳು
Ⓒ 6 ರಿಂದ 10 ಸಂದೇಶಗಳು
Ⓓ 10 ಕ್ಕಿಂತ ಜಾಸ್ತಿ ಸಂದೇಶಗಳು

11.5 ನೀವು ಕಳೆದ 30 ದಿನಗಳಲ್ಲಿ ದಿನಪತ್ರಿಕೆ/ಮ್ಯಾಗಜಿನ್‌ಗಳಲ್ಲಿ ಎಷ್ಟು ತಂಬಾಕು ವಿರೋಧಿ ಸಂದೇಶಗಳನ್ನು ನೋಡಿದ್ದೀರಾ?

Ⓐ ಒಂದೂ ಇಲ್ಲ
Ⓑ 1 ರಿಂದ 5 ಸಂದೇಶಗಳು
Ⓒ 6 ರಿಂದ 10 ಸಂದೇಶಗಳು
Ⓓ 10 ಕ್ಕಿಂತ ಜಾಸ್ತಿ ಸಂದೇಶಗಳು
Ⓔ ನಾನು ದಿನಪತ್ರಿಕೆ/ಮ್ಯಾಗಜಿನ್‌ಗಳನ್ನು ಓದುವುದಿಲ್ಲ

12. ಮುಂದಿನ ಪ್ರಶ್ನೆಗಳು ತಂಬಾಕು ಕುರಿತಾದ ಜಾಹೀರಾತುಗಳ ಬಗ್ಗೆ ಆಗಿವೆ

12.1 ಕಳೆದ 30 ದಿನಗಳಲ್ಲಿ, ಯಾವುದಾದರು ಸಾರ್ವಜನಿಕ ಸಾರಿಗೆ (ಬಸ್ಸು, ರೈಲು, ಟ್ಯಾಕ್ಸಿ, ಇತ್ಯಾದಿ) ಯಲ್ಲಿ ತಂಬಾಕು ಉತ್ಪನ್ನಗಳ ಬಗ್ಗೆ ಜಾಹೀರಾತುಗಳನ್ನು ಗಮನಿಸಿದ್ದೀರಾ?

Ⓐ ಹೌದು
Ⓑ ಇಲ್ಲ

12.2 ಕಳೆದ 30 ದಿನಗಳಲ್ಲಿ, ಸಾರ್ವಜನಿಕ ಸ್ಥಳಗಳಲ್ಲಿ ಯಾವುದಾದರು ತಂಬಾಕು ಉತ್ಪನ್ನಗಳ ಕುರಿತಾದ ಜಾಹೀರಾತುಗಳನ್ನು ಗೋಡೆ ಬರಹದಲ್ಲಿ ಗಮನಿಸಿದ್ದೀರಾ?

Ⓐ ಹೌದು
Ⓑ ಇಲ್ಲ

12.3 ಕಳೆದ 30 ದಿನಗಳಲ್ಲಿ, ಅಂತರ್ಜಾಲ(ಇಂಟರ್ನೆಟ್)ದಲ್ಲಿ ತಂಬಾಕು ಉತ್ಪನ್ನಗಳ ಬಗ್ಗೆ ಜಾಹೀರಾತುಗಳನ್ನು ಗಮನಿಸಿದ್ದೀರಾ?

Ⓐ ಹೌದು
Ⓑ ಇಲ್ಲ

12.4 ಕಳೆದ 30 ದಿನಗಳಲ್ಲಿ, ರೇಡಿಯೋ/ ದೂರದರ್ಶನದಲ್ಲಿ ತಂಬಾಕು ಉತ್ಪನ್ನಗಳ ಕುರಿತಾದ ಜಾಹೀರಾತುಗಳನ್ನು ಗಮನಿಸಿದ್ದೀರಾ?

Ⓐ ಹೌದು
Ⓑ ಇಲ್ಲ

12.5 ಕಳೆದ 30 ದಿನಗಳಲ್ಲಿ, ದಿನಪತ್ರಿಕೆ/ಮ್ಯಾಗಜಿನ್‌ಗಳಲ್ಲಿ ತಂಬಾಕು ಉತ್ಪನ್ನಗಳ ಬಗ್ಗೆ ಜಾಹೀರಾತುಗಳನ್ನು ಗಮನಿಸಿದ್ದೀರಾ?

Ⓐ ಹೌದು
Ⓑ ಇಲ್ಲ

13. ಈ ಕೆಳಗಿನ ವಾಕ್ಯಗಳನ್ನು ಓದಿ ಮತ್ತು ನಿಮ್ಮ ಮನೋಭಾವನೆಯನ್ನು ತಿಳಿಸಿ

13.1 ನನ್ನ ಪ್ರಕಾರ ನಾನು ಏನಾದರೂ ಮಾಡುವುದಾದರೆ, ನಾನು ಯಾವುದೇ ನಿಯಮಗಳನ್ನು ಪರಿಗಣಿಸುವುದಿಲ್ಲ

Ⓐ ಯಾವತ್ತೂ ಇಲ್ಲ
Ⓑ ಕೆಲವೊಮ್ಮೆ
Ⓒ ಯಾವಾಗಲೂ

13.2 ನನ್ನ ತಂದೆ ತಾಯಿಗೆ ಇಚ್ಛೆ ಇಲ್ಲದಿದ್ದರೂ, ನಾನು ಆ ಕೆಲಸವನ್ನು ಮಾಡುತ್ತೇನೆ

Ⓐ ಯಾವತ್ತೂ ಇಲ್ಲ
Ⓑ ಕೆಲವೊಮ್ಮೆ
Ⓒ ಯಾವಾಗಲೂ

13.3 ನಾನು ಶಾಲೆ, ಕೆಲಸ ಅಥವಾ ಇತರ ಸ್ಥಳಗಳಲ್ಲಿ ತೊಂದರೆಗೆ ಸಿಲುಕಿ ಕೊಳ್ಳುತ್ತೇನೆ

Ⓐ ಯಾವತ್ತೂ ಇಲ್ಲ
Ⓑ ಕೆಲವೊಮ್ಮೆ
Ⓒ ಯಾವಾಗಲೂ

14. ಈ ಮುಂದಿನ ಪ್ರಶ್ನೆಗಳು ನಿಮ್ಮ ಕುರಿತು ಮತ್ತು ಪರೀಕ್ಷೆಯಲ್ಲಿ ನಿಮ್ಮ ಸಾಧನೆ ಕುರಿತಾಗಿದೆ

14.1 ಈ ವಾಕ್ಯವನ್ನು ಓದಿ ಮತ್ತು ನಿಮಗೆ ಸರಿ ಅನಿಸಿದ ಆಯ್ಕೆಯನ್ನು ಗುರುತಿಸಿ. "ನನ್ನ ಆಲೋಚನೆ ಪ್ರಕಾರ ನನಗೆ ಹೆಚ್ಚಿನ ಸ್ವಾಭಿಮಾನವಿದೆ"

Ⓐ ದೃಢವಾಗಿ ಸಮ್ಮತಿಸುತ್ತೇನೆ
Ⓑ ಸಮ್ಮತಿಸುತ್ತೇನೆ
Ⓒ ಸಮ್ಮತಿ ಇಲ್ಲ, ಅಸಮ್ಮತಿಯೂ ಇಲ್ಲ
Ⓓ ಅಸಮ್ಮತಿಸುತ್ತೇನೆ
Ⓔ ದೃಢವಾಗಿ ಅಸಮ್ಮತಿಸುತ್ತೇನೆ

14.2 ಕಳೆದ ವಾರ್ಷಿಕ ಪರೀಕ್ಷೆಯಲ್ಲಿ ನಿಮ್ಮಸಾಧನೆಯ ಗುಣಮಟ್ಟವನ್ನು ತಿಳಿಸಿ

Ⓐ ಅತ್ಯುತ್ತಮ
Ⓑ ಉತ್ತಮ
Ⓒ ಸಾಧಾರಣ
Ⓓ ಸಾಧಾರಣಕ್ಕಿಂತ ಕಡಿಮೆ

15. ಕೊನೆಯ ಪ್ರಶ್ನೆ ನಿಮ್ಮ ಮನೆಗೆ ಸಂಬಂಧಿಸಿದ್ದಾಗಿರುತ್ತದೆ

15.1 ನಿಮ್ಮ ಮನೆಯಲ್ಲಿಇರುವ ವಸ್ತುಗಳು ಅಥವಾ ನಿಮ್ಮ ಮನೆಯಲ್ಲಿ ವಾಸವಾಗಿರುವ ವ್ಯಕ್ತಿಗಳಿಗೆ ಸೇರಿದ ವಸ್ತುಗಳಿಗೆ ಗುರುತು ಹಾಕಿ :

Ⓐ ವಿದ್ಯುತ್
Ⓑ ಫ್ಲಷ್ ಇರುವ ಶೌಚಾಲಯ
Ⓒ ಕಾರು
Ⓓ ಮೊಪೆಡ್/ಸ್ಕೂಟರ್/ಮೋಟಾರ್ ಸೈಕಲ್
Ⓔ ಟಿ.ವಿ
Ⓕ ಫ್ರಿಡ್ಜ್
Ⓖ ವಾಷಿಂಗ್ ಮೆಷಿನ್
Ⓗ ಲ್ಯಾಂಡ್ ಲೈನ್ ಫೋನ್
Ⓘ ಮೊಬೈಲ್ ಫೋನ್
Ⓙ ರೇಡಿಯೋ

ಈ ಪ್ರಶ್ನಾವಳಿಯನ್ನು ಭರ್ತಿಗೊಳಿಸಿದ್ದಕ್ಕಾಗಿ ಧನ್ಯವಾದಗಳು

ಸಂಶೋಧನಾ ಸಹಾಯಕ ಸಹಿ
