# Supplementary material for: Prospective cohort study of exposure to tobacco imagery in popular films and smoking uptake among children in southern India
Source: PLoS One. 2021 Aug 5;16(8):e0253593. doi: 10.1371/journal.pone.0253593 (PMC8341541; doi:10.1371/journal.pone.0253593)
Supplement: S1 File — (ZIP) [file pone.0253593.s002.zip › Year_Two_Questionneire_Kannada.pdf]

ಹದಿಹರೆಯದವರಲ್ಲಿ ಧೂಮಪಾನ ಸೇವನೆಯ ನಿಯಂತ್ರಣ–ಭಾರತದಲ್ಲಿ ದೀರ್ಘಕಾಲೀನ ಶ್ವಾಸಕೋಶದ ಖಾಯಿಲೆಯನ್ನು ತಡೆಗಟ್ಟುವ ಪ್ರಕ್ರಿಯೆಯ ಪ್ರಾಥಮಿಕ ಹಂತ

ಪ್ರಶ್ನಾವಳಿ – ಎರಡನೇ ವರ್ಷ

1. ಓ.ಎಮ್.ಆರ್ ಕ್ರಮ ಸಂಖ್ಯೆ

2.ವಲಯ ಸಂಖ್ಯೆ
- 1 ○
- 2 ○
- 3 ○
- 4 ○
- 5 ○

3. ಶಾಲಾ ಕೋಡ್ (6 columns, each 0 1 2 3 4 5 6 7 8 9)

4.ಶಾಲೆಯ ವಿಧ
- ಸರ್ಕಾರಿ ○
- ಅನುದಾನಿತ ○
- ಖಾಸಗಿ ○

5.ಪ್ರದೇಶ
- ನಗರ ○
- ಗ್ರಾಮೀಣ ○

6. ಹಾಜರಿ ಸಂಖ್ಯೆ (9 columns, each 0 1 2 3 4 5 6 7 8 9)

7. ಹುಟ್ಟಿದ ದಿನಾಂಕ

D D / M M / 2 0 Y Y

- D: 0 1 2 3 | D: 0 1 2 3 4 5 6 7 8 9
- M: 0 1 | M: 0 1 2 3 4 5 6 7 8 9
- Y: 0 1 | Y: 0 1 2 3 4 5 6 7 8 9

8. ಲಿಂಗ
- ಗಂಡು ○
- ಹೆಣ್ಣು ○

9. ತರಗತಿ
- 7 ○
- 8 ○
- 9 ○

10. ವಿಭಾಗ
- A ○
- B ○
- C ○
- D ○
- E ○
- F ○
- G ○
- H ○

11. ಧರ್ಮ
- ಹಿಂದೂ ○
- ಕ್ರಿಶ್ಚಿಯನ್ ○
- ಮುಸ್ಲಿಂ ○
- ಜೈನ್ ○
- ಇತರೆ ○

12. ತಂದೆಯ ಶಿಕ್ಷಣ
- ಅನಕ್ಷರಸ್ಥ ○
- ಓದಲು ಮಾತ್ರ ○
- 1-4 ○
- 5-7 ○
- 8-10 ○
- 11-12 ○
- ಡಿಪ್ಲೋಮ ○
- ಪದವಿ ○
- ಸ್ನಾತಕೋತ್ತರ ○
- ವೃತ್ತಿಪರ ○

13. ತಾಯಿಯ ಶಿಕ್ಷಣ
- ಅನಕ್ಷರಸ್ಥ ○
- ಓದಲು ಮಾತ್ರ ○
- 1-4 ○
- 5-7 ○
- 8-10 ○
- 11-12 ○
- ಡಿಪ್ಲೋಮ ○
- ಪದವಿ ○
- ಸ್ನಾತಕೋತ್ತರ ○
- ವೃತ್ತಿಪರ ○

14. ತಂದೆಯ ಉದ್ಯೋಗ		15. ತಾಯಿಯ ಉದ್ಯೋಗ	

ಗಮನಿಸಿ : ತಂಬಾಕು ಉತ್ಪನ್ನವು ತಂಬಾಕನ್ನು ಒಳಗೊಂಡ ವ್ಯಸನಕಾರಕ ವಸ್ತುವಾಗಿದೆ ಮತ್ತು ಇದರಲ್ಲಿ 2 ವಿಧದ ಉತ್ಪನ್ನಗಳಿವೆ :

ಹೊಗೆಸಹಿತ ತಂಬಾಕು – ಬೀಡಿ, ಸಿಗರೇಟ್, ಸಿಗಾರ್, ಚಿರೂಟ್ಸ್, ಸುರುಳಿ ಸಿಗರೇಟ್‌ಗಳು, ಯಾವುದೇ ತಂಬಾಕು ಮೆಕ್ಕೆಜೋಳದ ಎಲೆ ಅಥವಾ ವಾರ್ತಾ ಪತ್ರಿಕೆಯಲ್ಲಿ ಸುತ್ತಿಕೊಂಡಿರುವುದು, ಹುಕ್ಕಾ, ಪೈಪ್‌ಗಳು, ಚಿಲ್ಲಮ್, ಚುಟ್ಟಾ

ಹೊಗೆರಹಿತ ತಂಬಾಕು – ತಂಬಾಕು ಎಲೆ, ಅಡಿಕೆ ತುಂಡಿನ ಜೊತೆ ತಂಬಾಕು, ಸಾಡಾ/ಸುರ್ತಿ, ಖೈನಿ ಅಥವಾ ತಂಬಾಕು ಹುಳಿ ಮಿಶ್ರಣ, ಗುಟ್ಕ, ಜರ್ದಾ ಜೊತೆ ಪಾನ್ ಮಸಾಲ, ಗುಲ್, ಗುಡಾಕು, ಮಿಶ್ರಿ

ಕೆಲವು ಪ್ರಶ್ನೆಗಳು ಮುಖ್ಯವಾಗಿ ಹೊಗೆಸಹಿತ ತಂಬಾಕು ಅಥವಾ ಹೊಗೆರಹಿತ ತಂಬಾಕು ಉತ್ಪನ್ನದ ಬಗ್ಗೆ ಆಗಿವೆ. ಕೆಲವು ಪ್ರಶ್ನೆಗಳು 2 ವಿಧದ ತಂಬಾಕಿನ ಬಗ್ಗೆ ಸಾಮಾನ್ಯ ಪ್ರಶ್ನೆಗಳಾಗಿವೆ. ಪ್ರಶ್ನೆಗಳನ್ನು ಸರಿಯಾಗಿ ಓದಿ ಉತ್ತರಿಸಿ. ಸಂಶಯಗಳಿದ್ದರೆ ಸಿಬ್ಬಂದಿಗಳಲ್ಲಿ ಕೇಳಿ ಪರಿಹರಿಸಿಕೊಳ್ಳಿ.

1. ಈ ಪ್ರಶ್ನೆಗಳು ತಂಬಾಕಿನ ಪರಿಣಾಮಗಳ ತಿಳುವಳಿಕೆಯ ಬಗ್ಗೆ ಆಗಿವೆ

1.1 ತಂಬಾಕು ಬಳಕೆಯಿಂದ ಒಬ್ಬ ವ್ಯಕ್ತಿಯ ಆರೋಗ್ಯದ ಮೇಲೆ ಯಾವ ರೀತಿಯ ಪರಿಣಾಮ ಬೀರುತ್ತದೆ?
- Ⓐ ಉತ್ತಮ
- Ⓑ ಕೆಟ್ಟ ಪರಿಣಾಮ
- Ⓒ ಸರಿಯಾಗಿ ತಿಳಿದಿಲ್ಲ

1.2 ಯುವಜನತೆ ತಂಬಾಕಿನ ಬಳಕೆ ಮಾಡಿದರೆ ಆರೋಗ್ಯದ ಮೇಲೆ ಬಹು ಬೇಗನೆ ಕೆಟ್ಟ ಪರಿಣಾಮ ಬೀರುತ್ತದೆಯೇ?
- Ⓐ ಹೌದು
- Ⓑ ಇಲ್ಲ
- Ⓒ ಸರಿಯಾಗಿ ತಿಳಿದಿಲ್ಲ

1.3 ಕೇವಲ ಒಂದು ಅಥವಾ ಎರಡು ವರ್ಷಕ್ಕೆ ಮಾತ್ರ ಧೂಮಪಾನ ಅಥವಾ ತಂಬಾಕು ಸೇವನೆ ಮಾಡಿದರೆ ಸುರಕ್ಷಿತವೇ?
- Ⓐ ಹೌದು
- Ⓑ ಇಲ್ಲ
- Ⓒ ಸರಿಯಾಗಿ ತಿಳಿದಿಲ್ಲ

1.4 ನಿಮ್ಮ ಸುತ್ತಮುತ್ತಲಿನಲ್ಲಿ ಯಾರಾದರೂ ಧೂಮಪಾನ ಮಾಡಿದರೆ ಅದು ನಿಮ್ಮ ಆರೋಗ್ಯಕ್ಕೆ ಹಾನಿ ಉಂಟುಮಾಡುತ್ತದೆಯೇ?
- Ⓐ ಹೌದು
- Ⓑ ಇಲ್ಲ
- Ⓒ ಸರಿಯಾಗಿ ತಿಳಿದಿಲ್ಲ

1.5 ತಂಬಾಕು ಸೇವನೆಯನ್ನು ನಿಲ್ಲಿಸಿದರೆ ಅದು ವ್ಯಕ್ತಿಯ ಆರೋಗ್ಯವನ್ನು ಉತ್ತಮಗೊಳಿಸುತ್ತದೆಯೇ?
- Ⓐ ಹೌದು Ⓑ ಇಲ್ಲ
- Ⓒ ಗೊತ್ತಿಲ್ಲ

2. ಮುಂದಿನ ಪ್ರಶ್ನೆಗಳು ತಂಬಾಕು ವಿರೋಧಿ ನೀತಿಗಳ ಬಗ್ಗೆ ಆಗಿವೆ

2.1 ಸಾರ್ವಜನಿಕ ಸ್ಥಳಗಳಲ್ಲಿ ಧೂಮಪಾನ ಮಾಡುವುದನ್ನು ತಡೆಗಟ್ಟಲು ಕಾನೂನುಗಳಿವೆಯೇ?
- Ⓐ ಹೌದು
- Ⓑ ಇಲ್ಲ
- Ⓒ ಗೊತ್ತಿಲ್ಲ

2.2 ನಮ್ಮ ರಾಜ್ಯದಲ್ಲಿ ಹೊಗೆರಹಿತ ತಂಬಾಕನ್ನು(ಉದಾ: ಗುಟ್ಕ)ಜನರು ಮಾರಾಟ ಮಾಡುವುದನ್ನು ತಡೆಗಟ್ಟಲು ಕಾನೂನುಗಳಿವೆಯೇ?
- Ⓐ ಹೌದು
- Ⓑ ಇಲ್ಲ
- Ⓒ ಗೊತ್ತಿಲ್ಲ

2.3 ದೂರದರ್ಶನದ (ಟಿ.ವಿ.) ಚಾನಲ್‌ಗಳಲ್ಲಿ ಮತ್ತು ಪತ್ರಿಕಾ ಮಾಧ್ಯಮಗಳಲ್ಲಿ ತಂಬಾಕಿನ ಬಗ್ಗೆ ಜಾಹೀರಾತುಗಳನ್ನು ತಡೆಗಟ್ಟಲು ಕಾನೂನು ಇದೆಯೇ?
- Ⓐ ಹೌದು
- Ⓑ ಇಲ್ಲ
- Ⓒ ಗೊತ್ತಿಲ್ಲ

2.4 ನಿಮ್ಮ ಶಾಲೆಯಲ್ಲಿ "ಧೂಮಪಾನರಹಿತ ಪ್ರದೇಶ – ಧೂಮಪಾನ ಮಾಡುವುದು ಅಪರಾಧ" ಎಂಬ ಫಲಕ ಇದೆಯೇ?
- Ⓐ ಹೌದು Ⓑ ಇಲ್ಲ
- Ⓒ ಸರಿಯಾಗಿ ಗೊತ್ತಿಲ್ಲ

2.5 ನಿಮ್ಮ ಶಾಲೆಯ ಆವರಣದ ಮುಖ್ಯದ್ವಾರದ ಬಳಿ ಪ್ರಮುಖ ಪ್ರದೇಶದಲ್ಲಿ "ತಂಬಾಕು ಮುಕ್ತ ಶಾಲೆ" ಅಥವಾ "ತಂಬಾಕು ಮುಕ್ತ ಸಂಸ್ಥೆ" ಎಂಬ ಫಲಕ ಇದೆಯೇ?
- Ⓐ ಹೌದು Ⓑ ಇಲ್ಲ
- Ⓒ ಸರಿಯಾಗಿ ಗೊತ್ತಿಲ್ಲ

2.6 ತಂಬಾಕಿನ ಪೊಟ್ಟಣದ ಮೇಲೆ ತಂಬಾಕಿನ ದುಷ್ಪರಿಣಾಮಗಳ ಬಗ್ಗೆ ಇರುವ ಚಿತ್ರ ಅಥವಾ ಲಿಖಿತ ಎಚ್ಚರಿಕೆಯನ್ನು ಗಮನಿಸಿದ್ದೀರಾ?
- Ⓐ ಹೌದು
- Ⓑ ಇಲ್ಲ
- Ⓒ ನಾನು ಯಾವುದೇ ತಂಬಾಕಿನ ಪೊಟ್ಟಣ ನೋಡಿಲ್ಲ

2.7 ನಿಮ್ಮ ಶಾಲೆಯ ಹತ್ತಿರ ತಂಬಾಕು ಬಳಕೆ ಮಾಡುವುದನ್ನು ತೋರಿಸುವ ಜಾಹೀರಾತು ಫಲಕವನ್ನು ಗಮನಿಸಿದ್ದೀರಾ?
- Ⓐ ಹೌದು
- Ⓑ ಇಲ್ಲ

2.8 ಕಳೆದ 30 ದಿನಗಳಲ್ಲಿ, ನಿಮ್ಮ ಶಾಲೆಯ ಸುತ್ತಮುತ್ತ ಉಚಿತವಾಗಿ ತಂಬಾಕು ಉತ್ಪನ್ನಗಳ ಹಂಚಿಕೆ ಮಾಡುವುದನ್ನು ಗಮನಿಸಿದ್ದೀರಾ?
- Ⓐ ಹೌದು
- Ⓑ ಇಲ್ಲ

2.9 ಕಳೆದ 30 ದಿನಗಳಲ್ಲಿ, ನಿಮ್ಮ ಶಾಲಾ ವಠಾರದ 100 ಯಾರ್ಡ್(90 ಮೀಟರ್) ಅಂತರದಲ್ಲಿ ತಂಬಾಕು ಉತ್ಪನ್ನಗಳ ಮಾರಾಟ ಮಾಡುವುದನ್ನು ಗಮನಿಸಿದ್ದೀರಾ?
- Ⓐ ಹೌದು
- Ⓑ ಇಲ್ಲ

3. ಈ ಮುಂದಿನ ಪ್ರಶ್ನೆಗಳು ಅಂಗಡಿಗಳ ಕುರಿತಾಗಿದೆ

3.1 ನೀವು ಸೂಪರ್ ಮಾರ್ಕೆಟ್‌ಗಳಿಗೆ ಹೋದಾಗ ತಂಬಾಕು ಉತ್ಪನ್ನಗಳನ್ನು ಮಾರಾಟ ಮಾಡಲು ಇಟ್ಟಿರುವುದನ್ನು ಎಷ್ಟು ಸಲ ಗಮನಿಸಿದ್ದೀರಿ ?
- Ⓐ ಪ್ರತಿಸಲ
- Ⓑ ಹೆಚ್ಚಿನ ಸಲ
- Ⓒ ಕೆಲವು ಸಲ
- Ⓓ ಯಾವಾಗಲಾದರೊಮ್ಮೆ
- Ⓔ ಯಾವತ್ತೂ ಇಲ್ಲ
- Ⓕ ನಾನು ಸೂಪರ್ ಮಾರ್ಕೆಟ್‌ಗಳಿಗೆ ಹೋಗುವುದಿಲ್ಲ

3.2 ನೀವು ಸಣ್ಣ ಅಂಗಡಿಗಳಿಗೆ(ಸಣ್ಣ ದಿನಸಿ ಅಂಗಡಿಗಳು, ಪಾನ್‌ಶಾಪ್‌ಗಳು) ಹೋದಾಗ ಎಷ್ಟು ಸಲ ತಂಬಾಕು ಉತ್ಪನ್ನಗಳನ್ನು ಮಾರಲು ಇಟ್ಟಿರುವುದನ್ನು ಗಮನಿಸಿದ್ದೀರಿ ?
- Ⓐ ಪ್ರತಿಸಲ
- Ⓑ ಹೆಚ್ಚಿನ ಸಲ
- Ⓒ ಕೆಲವು ಸಲ
- Ⓓ ಯಾವಾಗಲಾದರೊಮ್ಮೆ
- Ⓔ ಯಾವತ್ತೂ ಇಲ್ಲ
- Ⓕ ನಾನು ಸಣ್ಣಅಂಗಡಿಗಳಿಗೆ ಹೋಗುವುದಿಲ್ಲ

3.3 ನೀವು ಸೂಪರ್ ಮಾರ್ಕೆಟ್ ಅಥವಾ ಸಣ್ಣ ಅಂಗಡಿಗಳಿಗೆ ಹೋದಾಗ ಯಾವುದೇ ರೀತಿಯ ತಂಬಾಕಿನ ಬ್ರಾಂಡ್‌ಗಳನ್ನು ಪ್ರದರ್ಶನಕ್ಕೆ ಇಟ್ಟಿರುವುದನ್ನು ಗಮನಿಸಿದ್ದೀರಾ?
- Ⓐ ಹೌದು, ನಾನು ಗಮನಿಸಿದ್ದೇನೆ
  ನಿಮ್ಮ ಉತ್ತರ ಹೌದಾದಲ್ಲಿ, ಬ್ರಾಂಡ್‌ನ್ನು ತಿಳಿಸಿ______________________
  ____
- Ⓑ ಇಲ್ಲ, ನಾನು ಯಾವುದೇ ಬ್ರಾಂಡ್‌ಗಳನ್ನು ಪ್ರದರ್ಶನಕ್ಕೆ ಇಟ್ಟಿರುವುದನ್ನು ಯಾವತ್ತು ನೋಡಿಲ್ಲ
- Ⓒ ನನಗೆ ಯಾವುದೇ ಬ್ರಾಂಡ್‌ಗಳು ನೆನೆಪಿನಲ್ಲಿ ಇಲ್ಲ
- Ⓓ ನಾನು ಅಂಗಡಿ/ಸೂಪರ್ ಮಾರ್ಕೆಟ್‌ಗಳಿಗೆ ಹೋಗುವುದಿಲ್ಲ

3.4 ಒಂದು ವೇಳೆ ನಿಮ್ಮ ಪ್ರಾಯದವರು ಅಂಗಡಿಗಳಲ್ಲಿ ತಂಬಾಕು ಉತ್ಪನ್ನಗಳನ್ನು ಖರೀದಿಸಲು ಪ್ರಯತ್ನಿಸಿದಲ್ಲಿ, ನಿಮ್ಮ ಪ್ರಕಾರ ಅವರು ಸಫಲರಾಗುತ್ತಾರೆಯೇ ?
- Ⓐ ಹೌದು
- Ⓑ ಇಲ್ಲ
- Ⓒ ಗೊತ್ತಿಲ್ಲ

4. ಮುಂದಿನ ಪ್ರಶ್ನೆಗಳು ಧೂಮಪಾನದ ಬಗ್ಗೆಯಾಗಿದೆ

4.1 ನಿಮ್ಮ ಮನೆಯಲ್ಲಿ ಧೂಮಪಾನ ಬಳಕೆಗೆ ಅವಕಾಶವಿದೆಯೇ?
- Ⓐ ಹೌದು
- Ⓑ ಇಲ್ಲ

4.2 ನಿಮ್ಮ ಕುಟುಂಬದಲ್ಲಿ ಯಾರಾದರೂ ಧೂಮಪಾನ ಬಳಸುತ್ತಾರೆಯೇ (ನಿಮಗೆ ಅನ್ವಯಿಸುವ ಎಲ್ಲಾ ವೃತ್ತಗಳಲ್ಲಿ ಗುರುತಿಸಿ)
- Ⓐ ಯಾರೂ ಇಲ್ಲ
- Ⓑ ತಾಯಿ
- Ⓒ ತಂದೆ
- Ⓓ ಸಹೋದರ
- Ⓔ ಸಹೋದರಿ
- Ⓕ ಇತರ

4.3 ನಿಮ್ಮ ಎಷ್ಟು ಸ್ನೇಹಿತರು ಧೂಮಪಾನ ಮಾಡುತ್ತಾರೆ?
- Ⓐ ಯಾರೂ ಇಲ್ಲ
- Ⓑ ಒಬ್ಬ
- Ⓒ ಇಬ್ಬರೂ
- Ⓓ ಮೂವರು ಅಥವಾ ಹೆಚ್ಚು
- Ⓔ ಸರಿಯಾಗಿ ಗೊತ್ತಿಲ್ಲ

4.4 ಶಾಲಾ ಕಟ್ಟಡ ಅಥವಾ ಶಾಲಾ ವಠಾರದಲ್ಲಿ ಯಾರಾದರೂ ಧೂಮಪಾನ ಮಾಡುವುದನ್ನು ನೋಡಿದ್ದೀರಾ?
- Ⓐ ಹೌದು
- Ⓑ ಇಲ್ಲ

4.5 ಈ ಕೆಳಗಿನ ಹೇಳಿಕೆಗಳನ್ನು ಗಮನವಿಟ್ಟು ಓದಿ ಮತ್ತು ನಿಮ್ಮ ಕುರಿತು ಧೂಮಪಾನದ ಬಗ್ಗೆ ಅನ್ವಯವಾಗುವ ಆಯ್ಕೆಯನ್ನು ಗುರುತಿಸಿ.
- Ⓐ ನಾನು ಯಾವತ್ತು ಧೂಮಪಾನ ಮಾಡಲಿಲ್ಲ
- Ⓑ ನಾನು ಹಿಂದೆ ಧೂಮಪಾನ ಮಾಡಿದ್ದೆ, ಆದರೆ ಕಳೆದ 30 ದಿನಗಳಲ್ಲಿ ಮಾಡಿಲ್ಲ
- Ⓒ ನಾನು ಕೆಲವೊಮ್ಮೆ ಧೂಮಪಾನ ಮಾಡುತ್ತೇನೆ, ಆದರೆ ವಾರಕ್ಕೆ 1 ಬಾರಿಗಿಂತ ಕಡಿಮೆ
- Ⓓ ನಾನು ವಾರದಲ್ಲಿ 1 ರಿಂದ 6 ಬಾರಿ ಧೂಮಪಾನ ಮಾಡುತ್ತೇನೆ
- Ⓔ ನಾನು ವಾರದಲ್ಲಿ 6ಕ್ಕಿಂತ ಹೆಚ್ಚಿನ ಬಾರಿ ಧೂಮಪಾನ ಮಾಡುತ್ತೇನೆ

4.6 ಪ್ರಥಮ ಬಾರಿಗೆ ಧೂಮಪಾನ ಪ್ರಯತ್ನಿಸಿದಾಗ ನಿಮ್ಮ ಪ್ರಾಯ?
- Ⓐ ನಾನು ಧೂಮಪಾನ ಮಾಡಲು ಪ್ರಯತ್ನಿಸಿಲ್ಲ
- Ⓑ 7 ವರ್ಷ ಅಥವಾ ಕಡಿಮೆ ಪ್ರಾಯ
- Ⓒ 8 ವರ್ಷ ಪ್ರಾಯ
- Ⓓ 9 ವರ್ಷ ಪ್ರಾಯ
- Ⓔ 10 ವರ್ಷ ಪ್ರಾಯ
- Ⓕ 11 ವರ್ಷ ಪ್ರಾಯ
- Ⓖ 12 ವರ್ಷ ಪ್ರಾಯ
- Ⓗ 13 ವರ್ಷ ಪ್ರಾಯ
- Ⓘ 14 ವರ್ಷ ಪ್ರಾಯ
- Ⓙ 15 ವರ್ಷ ಅಥವಾ ಹೆಚ್ಚಿನ ಪ್ರಾಯ

4.7 ನೀವು ಮೊದಲ ಬಾರಿ ಯಾವ ವಿಧದ ಧೂಮಪಾನ ಮಾಡಿದ್ದೀರಿ?
- Ⓐ ನಾನು ಯಾವತ್ತೂ ಧೂಮಪಾನ ಮಾಡಿಲ್ಲ
- Ⓑ ನಾನು ಸಿಗರೇಟ್ ಸೇದಿದ್ದೇನೆ
- Ⓒ ನಾನು ಬೀಡಿ ಸೇದಿದ್ದೇನೆ
- Ⓓ ನಾನು ಹುಕ್ಕಾ ಸೇದಿದ್ದೇನೆ
- Ⓔ ಬೇರೆ ಯಾವುದಾದರೆ, ತಿಳಿಸಿ___________________________

4.8 ನೀವು ಧೂಮಪಾನ ಆರಂಭಿಸಲು ಮುಖ್ಯ ಕಾರಣವೇನು?
- Ⓐ ನಾನು ಯಾವತ್ತೂ ಧೂಮಪಾನ ಮಾಡಿಲ್ಲ
- Ⓑ ಒಂಟಿತನ
- Ⓒ ಸ್ನೇಹಿತರ ಒತ್ತಾಯ
- Ⓓ ಹಿರಿಯರಿಂದ ಕಲಿತೆ
- Ⓔ ಕುತೂಹಲ
- Ⓕ ಪ್ರೌಢರಾಗಿ ಕಾಣಲು
- Ⓖ ಒತ್ತಡ
- Ⓗ ಹೆಚ್ಚು ಸ್ನೇಹಿತರನ್ನು ಹೊಂದಲು
- Ⓘ ಆಕರ್ಷಕವಾಗಿ ಕಾಣಲು
- Ⓙ ನಟ/ನಟಿಯರ ಧೂಮಪಾನ ಮಾಡುವುದು
- Ⓚ ಇತರೆ ಕಾರಣಗಳಿದ್ದರೆ, ತಿಳಿಸಿ: ______________________________

4.9 ನೀವು ಮೊದಲ ಬಾರಿ ಸಿಗರೇಟ್/ಬೀಡಿಗಳನ್ನು ಯಾವ ರೀತಿಯಲ್ಲಿ ಪಡೆದುಕೊಂಡಿರಿ?
- Ⓐ ನಾನು ಧೂಮಪಾನದ ಬಳಕೆ ಮಾಡಿಲ್ಲ
- Ⓑ ನಾನು ಅಂಗಡಿಯಲ್ಲಿ ಖರೀದಿಸಿದೆ
- Ⓒ ನಾನು ಇಂಟರ್ನೆಟ್/ಆನ್‌ಲೈನ್ ಮೂಲಕ ಪಡೆದುಕೊಂಡೆ
- Ⓓ ನಾನು ಬೇರೆಯವರಿಗೆ ಹಣಕೊಟ್ಟು ನನಗೋಸ್ಕರ ಖರೀದಿಸಿದೆ
- Ⓔ ನಾನು ಇನ್ನೊಬ್ಬರಿಂದ ಕೇಳಿ ಪಡೆದುಕೊಂಡೆ
- Ⓕ ಕುಟುಂಬದ ಸದಸ್ಯರಿಂದ ಪಡೆದುಕೊಂಡೆ
- Ⓖ ನಾನು ಭಾಗಶ: ಉರಿದ ಸಿಗರೇಟ್/ಬೀಡಿಯನ್ನು ಸೇದಿದೆ
- Ⓗ ಬೇರೆ ಇದ್ದಲ್ಲಿ, ತಿಳಿಸಿ________________________________

5. ಮುಂದಿನ ಪ್ರಶ್ನೆಗಳು ಒಂದು ತಿಂಗಳ (30 ದಿನಗಳ) ಹಿಂದಿನ ಧೂಮಪಾನದ ಬಗ್ಗೆಯಾಗಿದೆ

5.1 ನೀವು ಕಳೆದ 30 ದಿನಗಳಲ್ಲಿ ಎಷ್ಟು ಬಾರಿ ಧೂಮಪಾನ ಮಾಡಿದ್ದೀರಿ?
- Ⓐ ನಾನು ಧೂಮಪಾನದ ಬಳಕೆ ಮಾಡಿಲ್ಲ
- Ⓑ ವಾರದಲ್ಲಿ ಒಂದು ಸಲಕ್ಕಿಂತ ಕಡಿಮೆ
- Ⓒ ವಾರದಲ್ಲಿ ಒಂದರಿಂದ ಮೂರು ಸಲ
- Ⓓ ವಾರದಲ್ಲಿ ನಾಲ್ಕರಿಂದ ಆರು ಸಲ
- Ⓔ ವಾರದಲ್ಲಿ ಆರು ಸಲಕ್ಕಿಂತ ಜಾಸ್ತಿ

5.2 ನೀವು ಕಳೆದ 30 ದಿನಗಳಲ್ಲಿ (ಒಂದು ತಿಂಗಳು) ನಿಮಗೋಸ್ಕರ ಎಷ್ಟು ಸಿಗರೇಟ್‌ಗಳನ್ನು ಖರೀದಿಸಿದ್ದೀರಿ?
- Ⓐ ಒಂದೂ ಇಲ್ಲ
- Ⓑ ಒಂದು ಸಿಗರೇಟ್
- Ⓒ 2-9 ಸಿಗರೇಟ್
- Ⓓ 10 ಸಿಗರೇಟ್‌ಗಳ ಒಂದು ಪ್ಯಾಕ್
- Ⓔ 20 ಸಿಗರೇಟ್‌ಗಳ ಒಂದು ಪ್ಯಾಕ್
- Ⓕ 20ಕ್ಕಿಂತ ಹೆಚ್ಚು ಸಿಗರೇಟ್‌ಗಳು

5.3 ಕಳೆದ 30 ದಿನಗಳಲ್ಲಿ ನಿಮಗೋಸ್ಕರ ಸೇದುವ ಸಿಗರೇಟಿಗೆ ಎಷ್ಟು ಹಣ ಖರ್ಚು ಮಾಡಿದ್ದೀರಿ?
- Ⓐ ಏನೂ ಖರ್ಚು ಮಾಡಿಲ್ಲ
- Ⓑ ನಾನು ಸೇದುತ್ತೇನೆ ಆದರೆ ಖರೀದಿಸುವುದಿಲ್ಲ
- Ⓒ 10 ರೂ. ಗಿಂತ ಕಡಿಮೆ
- Ⓓ ರೂ. 11 ರಿಂದ 30
- Ⓔ ರೂ. 31 ರಿಂದ 60
- Ⓕ ರೂ. 61 ರಿಂದ 100
- Ⓖ ರೂ. 100 ಕ್ಕಿಂತ ಹೆಚ್ಚು

5.4 ನೀವು ಕಳೆದ 30 ದಿನಗಳಲ್ಲಿ (ಒಂದು ತಿಂಗಳು) ನಿಮಗೋಸ್ಕರ ಎಷ್ಟು ಬೀಡಿಗಳನ್ನು ಖರೀದಿಸಿದ್ದೀರಿ?
- Ⓐ ಒಂದೂ ಇಲ್ಲ
- Ⓑ ಒಂದು ಬೀಡಿ
- Ⓒ 2-5 ಬೀಡಿಗಳು
- Ⓓ 6-10 ಬೀಡಿಗಳು
- Ⓔ 11-20 ಬೀಡಿಗಳು
- Ⓕ 25 ಬೀಡಿಗಳ ಒಂದು ಪ್ಯಾಕ್

5.5 ಕಳೆದ 30 ದಿನಗಳಲ್ಲಿ ನಿಮಗೋಸ್ಕರ ಸೇದುವ ಬೀಡಿಗಳಿಗೆ ಎಷ್ಟು ಹಣ ಖರ್ಚು ಮಾಡಿದ್ದೀರಿ?
- Ⓐ ಏನೂ ಖರ್ಚು ಮಾಡಿಲ್ಲ
- Ⓑ ನಾನು ಸೇದುತ್ತೇನೆ ಆದರೆ ಖರೀದಿಸಿಲ್ಲ
- Ⓒ 10 ರೂ. ಗಿಂತ ಕಡಿಮೆ
- Ⓓ ರೂ. 11 ರಿಂದ 20
- Ⓔ ರೂ. 21 ರಿಂದ 30
- Ⓕ ರೂ. 30 ಕ್ಕಿಂತ ಹೆಚ್ಚು

5.6 ನೀವು ಕಳೆದ 07 ದಿನಗಳಲ್ಲಿ ಎಷ್ಟು ಸಿಗರೇಟ್‌ಗಳನ್ನು ಸೇದಿದ್ದೀರಿ?
- Ⓐ ಸೇದಲೇ ಇಲ್ಲ
- Ⓑ 1-2
- Ⓒ 3-5
- Ⓓ 6 ಕ್ಕಿಂತ ಜಾಸ್ತಿ

5.7 ನೀವು ಕಳೆದ 07 ದಿನಗಳಲ್ಲಿ ಎಷ್ಟು ಬೀಡಿಗಳನ್ನು ಸೇದಿದ್ದೀರಿ?
- Ⓐ ಸೇದಲೇ ಇಲ್ಲ
- Ⓑ 1-2
- Ⓒ 3-5
- Ⓓ 6 ಕ್ಕಿಂತ ಜಾಸ್ತಿ

6. ಮುಂದೆ ಬರುವ ಪ್ರಶ್ನೆಗಳು ಧೂಮಪಾನದ ಬಳಕೆಯ ಬಗ್ಗೆ ನಿಮ್ಮ ಯೋಜನೆಗಳು ಮತ್ತು ಆಲೋಚನೆಗಳ ಬಗ್ಗೆ ಆಗಿವೆ

6.1 ಶೀಘ್ರದಲ್ಲಿ ನೀವು ಧೂಮಪಾನ ಮಾಡಲು ಆರಂಭಿಸುತ್ತೀರಿ ಎಂದು ನಿಮಗೆ ಅನಿಸುತ್ತದೆಯೇ?

Ⓐ ಹೌದು
Ⓑ ಇಲ್ಲ

6.2 ನಿಮ್ಮ ಆತ್ಮೀಯ ಸ್ನೇಹಿತರಲ್ಲಿ ಒಬ್ಬ ನಿಮಗೆ ಸಿಗರೇಟ್ ನೀಡಿದಲ್ಲಿ ನೀವು ಅದನ್ನು ಬಳಸುತ್ತೀರಾ?

Ⓐ ಖಂಡಿತವಾಗಿಯೂ ಹೌದು Ⓑ ಪ್ರಾಯಶ: ಹೌದು
Ⓒ ಪ್ರಾಯಶ: ಇಲ್ಲ Ⓓ ಖಂಡಿತವಾಗಿಯೂ ಇಲ್ಲ

6.3 ನೀವು ಮುಂದಿನ ವರ್ಷ ಯಾವತ್ತಾದರೂ ಧೂಮಪಾನ ಮಾಡಬಹುದು ಎಂದು ಭಾವಿಸುತ್ತೀರಾ?

Ⓐ ಖಂಡಿತವಾಗಿಯೂ ಹೌದು Ⓑ ಪ್ರಾಯಶ: ಹೌದು
Ⓒ ಪ್ರಾಯಶ: ಇಲ್ಲ Ⓓ ಖಂಡಿತವಾಗಿಯೂ ಇಲ್ಲ

6.4 ಮುಂದೆ ನೀವು ಕಾಲೇಜಿಗೆ ಹೋಗಲು ಪ್ರಾರಂಭಿಸಿದಾಗ ಧೂಮಪಾನ ಮಾಡುವ ಆಲೋಚನೆ ಇದೆಯೇ?

Ⓐ ಖಂಡಿತವಾಗಿಯೂ ಹೌದು Ⓑ ಪ್ರಾಯಶ: ಹೌದು
Ⓒ ಪ್ರಾಯಶ: ಇಲ್ಲ Ⓓ ಖಂಡಿತವಾಗಿಯೂ ಇಲ್ಲ

7. ಮುಂದಿನ ಪ್ರಶ್ನೆಗಳು ಹೊಗೆರಹಿತ ತಂಬಾಕು ಬಳಕೆಯ ಬಗ್ಗೆಯಾಗಿದೆ
(ಜಗಿಯುವ ತಂಬಾಕು, ಗುಟ್ಕಾ, ಖೈನಿ, ಜರ್ದಾ, ನಶ್ಯ)

7.1 ನಿಮ್ಮ ಮನೆಯಲ್ಲಿ ಹೊಗೆರಹಿತ ತಂಬಾಕಿನ ಬಳಕೆಗೆ ಅವಕಾಶವಿದೆಯೇ ?

Ⓐ ಹೌದು
Ⓑ ಇಲ್ಲ

7.2 ನಿಮ್ಮ ಕುಟುಂಬದಲ್ಲಿ ಯಾರಾದರೂ ಹೊಗೆರಹಿತ ತಂಬಾಕು ಬಳಸುತ್ತಾರೆಯೇ? (ನಿಮಗೆ ಅನ್ವಯಿಸುವ ಎಲ್ಲಾ ವೃತ್ತಗಳಲ್ಲಿ ಗುರುತಿಸಿ)

Ⓐ ಯಾರೂ ಇಲ್ಲ Ⓑ ತಾಯಿ
Ⓒ ತಂದೆ Ⓓ ಸಹೋದರ
Ⓔ ಸಹೋದರಿ Ⓕ ಇತರ

7.3 ನಿಮ್ಮ ಎಷ್ಟು ಸ್ನೇಹಿತರು ಹೊಗೆರಹಿತ ತಂಬಾಕು ಬಳಸುತ್ತಾರೆ?

Ⓐ ಯಾರೂ ಇಲ್ಲ Ⓑ ಒಬ್ಬ
Ⓒ ಇಬ್ಬರೂ Ⓓ ಮೂವರು ಅಥವಾ ಹೆಚ್ಚು
Ⓔ ಸರಿಯಾಗಿ ಗೊತ್ತಿಲ್ಲ

7.4 ಈ ಕೆಳಗಿನ ಹೇಳಿಕೆಗಳನ್ನು ಗಮನವಿಟ್ಟು ಓದಿ ಮತ್ತು ನಿಮ್ಮ ಕುರಿತು ಹೊಗೆರಹಿತ ತಂಬಾಕಿನ ಬಗ್ಗೆ ಅನ್ವಯವಾಗುವ ಆಯ್ಕೆಯನ್ನು ಗುರುತಿಸಿ.

Ⓐ ನಾನು ಯಾವತ್ತು ಹೊಗೆರಹಿತ ತಂಬಾಕಿನ ಬಳಕೆ ಮಾಡಲಿಲ್ಲ
Ⓑ ನಾನು ಹಿಂದೆ ಹೊಗೆರಹಿತ ತಂಬಾಕು ಬಳಸಿದ್ದೇನೆ, ಆದರೆ ಕಳೆದ 30ದಿನಗಳಲ್ಲಿ ಇಲ್ಲ
Ⓒ ನಾನು ಕೆಲವೊಮ್ಮೆ ಹೊಗೆರಹಿತ ತಂಬಾಕು ಬಳಸುತ್ತೇನೆ, ಆದರೆ ವಾರಕ್ಕೆ 1 ಬಾರಿಗಿಂತ ಕಡಿಮೆ
Ⓓ ನಾನು ವಾರದಲ್ಲಿ 1 ರಿಂದ 6 ಬಾರಿ ಹೊಗೆರಹಿತ ತಂಬಾಕು ಬಳಸುತ್ತೇನೆ.
Ⓔ ನಾನು ವಾರದಲ್ಲಿ 6 ಕ್ಕಿಂತ ಹೆಚ್ಚಿನ ಬಾರಿ ಹೊಗೆರಹಿತ ತಂಬಾಕು ಬಳಸುತ್ತೇನೆ.

7.5 ಪ್ರಥಮ ಬಾರಿ ಹೊಗೆರಹಿತ ತಂಬಾಕಿನ ಬಳಕೆ ಪ್ರಯತ್ನಿಸಿದಾಗ ನಿಮ್ಮ ಪ್ರಾಯ?

Ⓐ ನಾನು ಪ್ರಯತ್ನಿಸಿಲ್ಲ Ⓑ 7 ವರ್ಷ ಅಥವಾ ಕಡಿಮೆ ಪ್ರಾಯ
Ⓒ 8 ವರ್ಷ ಪ್ರಾಯ Ⓓ 9 ವರ್ಷ ಪ್ರಾಯ
Ⓔ 10 ವರ್ಷ ಪ್ರಾಯ Ⓕ 11 ವರ್ಷ ಪ್ರಾಯ
Ⓖ 12 ವರ್ಷ ಪ್ರಾಯ Ⓗ 13 ವರ್ಷ ಪ್ರಾಯ
Ⓘ 14 ವರ್ಷ ಪ್ರಾಯ Ⓙ 15 ವರ್ಷ ಅಥವಾ ಹೆಚ್ಚಿನ ಪ್ರಾಯ

7.6 ನೀವು ಮೊದಲ ಬಾರಿ ಹೊಗೆರಹಿತ ತಂಬಾಕಿನ ಬಳಕೆ ಆರಂಭಿಸಲು ಮುಖ್ಯ ಕಾರಣವೇನು?

Ⓐ ನಾನು ಬಳಕೆ ಮಾಡಿಲ್ಲ Ⓑ ಒಂಟಿತನ
Ⓒ ಸ್ನೇಹಿತರ ಒತ್ತಾಯ Ⓓ ಹಿರಿಯರಿಂದ ಕಲಿತೆ
Ⓔ ಕುತೂಹಲ Ⓕ ಪ್ರೌಢರಾಗಿ ಕಾಣಲು
Ⓖ ಒತ್ತಡ Ⓗ ಹೆಚ್ಚು ಸ್ನೆಹಿತರನ್ನು ಹೊಂದಲು
Ⓘ ರುಚಿಯನ್ನು ಆಸ್ವಾಧಿಸಲು Ⓙ ಆಕರ್ಷಿತರಾಗಿ ಕಾಣಲು
Ⓚ ಇತರೆ ಕಾರಣ ಇದ್ದಲ್ಲಿ ತಿಳಿಸಿ:___________________________

7.7 ನೀವು ಕಳೆದ 30 ದಿನಗಳಲ್ಲಿ, ಹೊಗೆರಹಿತ ತಂಬಾಕಿನ ಬಳಕೆ ಮಾಡಿದ್ದಲ್ಲಿ, ಈ ಕೆಳಗಿನವುಗಳಲ್ಲಿ ಯಾವುದನ್ನು ಬಳಸಿದ್ದೀರಿ? (ನಿಮಗೆ ಅನ್ವಯಿಸುವ ಎಲ್ಲಾ ವೃತ್ತಗಳಲ್ಲಿ ಗುರುತಿಸಿ)

Ⓐ ನಾನು ಬಳಕೆ ಮಾಡಿಲ್ಲ Ⓑ ನಶ್ಯ
Ⓒ ಖೈನಿ Ⓓ ಜಗಿಯುವ ತಂಬಾಕು
Ⓔ ಗುಟ್ಕಾ Ⓕ ಜರ್ದಾ
Ⓖ ಇತರೆ, ಇದ್ದಲ್ಲಿ ತಿಳಿಸಿ:_______________________________

7.8 ನೀವು ಕಳೆದ 30 ದಿನಗಳಲ್ಲಿ (ಒಂದು ತಿಂಗಳು), ನಿಮಗೋಸ್ಕರ ಎಷ್ಟು ಹೊಗೆರಹಿತ ತಂಬಾಕನ್ನು ಖರೀದಿಸಿದ್ದೀರಿ?

Ⓐ ಇಲ್ಲ, ನಾನು ಬಳಕೆ ಮಾಡಿಲ್ಲ Ⓑ ನಾನು ಬಳಸುತ್ತೇನೆ ಆದರೆ ಖರೀದಿಸಿಲ್ಲ
Ⓒ ಒಂದು ಪ್ಯಾಕೆಟ್ Ⓓ 2-5 ಪ್ಯಾಕೆಟ್
Ⓔ 5-10 ಪ್ಯಾಕೆಟ್ Ⓕ 10ಕ್ಕಿಂತ ಹೆಚ್ಚು ಪ್ಯಾಕೆಟ್‌ಗಳು

7.9 ನೀವು ಕಳೆದ 7 ದಿನಗಳಲ್ಲಿ ಎಷ್ಟು ಪ್ಯಾಕೆಟ್ ಹೊಗೆರಹಿತ ತಂಬಾಕನ್ನು ಜಗಿದಿದ್ದೀರಿ?

Ⓐ ಇಲ್ಲ ನಾನು ಬಳಕೆ ಮಾಡಿಲ್ಲ Ⓑ ಒಂದು ಪ್ಯಾಕೆಟ್
Ⓒ 2-5 ಪ್ಯಾಕೆಟ್ Ⓓ 6-10 ಪ್ಯಾಕೆಟ್‌ಗಳು
Ⓔ 10ಕ್ಕಿಂತ ಹೆಚ್ಚು ಪ್ಯಾಕೆಟ್‌ಗಳು

8. ಮುಂದಿನ ಪ್ರಶ್ನೆಗಳು ಹೊಗೆರಹಿತ ತಂಬಾಕಿನ ಬಳಕೆಯ ಬಗ್ಗೆ ನಿಮ್ಮ ಯೋಜನೆಗಳು ಮತ್ತು ಆಲೋಚನೆಗಳ ಬಗ್ಗೆ ಆಗಿವೆ

8.1 ಶೀಘ್ರದಲ್ಲಿ ನೀವು ಹೊಗೆರಹಿತ ತಂಬಾಕು ಬಳಸಲು ಆರಂಭಿಸುತ್ತೀರಿ ಎಂದು ನಿಮಗೆ ಅನಿಸುತ್ತದೆಯೇ?

Ⓐ ಹೌದು
Ⓑ ಇಲ್ಲ

8.2 ನಿಮ್ಮ ಆತ್ಮೀಯ ಸ್ನೇಹಿತರಲ್ಲಿ ಒಬ್ಬ ನಿಮಗೆ ಹೊಗೆರಹಿತ ತಂಬಾಕು ಕೊಟ್ಟಲ್ಲಿ ನೀವು ಬಳಸುತ್ತೀರಾ?

Ⓐ ಖಂಡಿತವಾಗಿಯೂ ಹೌದು Ⓑ ಪ್ರಾಯಶ: ಹೌದು
Ⓒ ಪ್ರಾಯಶ: ಇಲ್ಲ Ⓓ ಖಂಡಿತವಾಗಿಯೂ ಇಲ್ಲ

8.3 ನೀವು ಮುಂದಿನ ವರ್ಷ ಯಾವತ್ತಾದರೂ ಹೊಗೆರಹಿತ ತಂಬಾಕು ಬಳಸಬಹುದೆಂದು ಭಾವಿಸುತ್ತೀರಾ?

Ⓐ ಖಂಡಿತವಾಗಿಯೂ ಹೌದು Ⓑ ಪ್ರಾಯಶ: ಹೌದು
Ⓒ ಪ್ರಾಯಶ: ಇಲ್ಲ Ⓓ ಖಂಡಿತವಾಗಿಯೂ ಇಲ್ಲ

8.4 ಮುಂದೆ ನೀವು ಕಾಲೇಜಿಗೆ ಹೋಗಲು ಪ್ರಾರಂಭಿಸಿದಾಗ ಹೊಗೆರಹಿತ ತಂಬಾಕಿನ ಬಳಕೆ ಮಾಡುವ ಆಲೋಚನೆ ಇದೆಯೇ?

Ⓐ ಖಂಡಿತವಾಗಿಯೂ ಹೌದು Ⓑ ಪ್ರಾಯಶ: ಹೌದು
Ⓒ ಪ್ರಾಯಶ: ಇಲ್ಲ Ⓓ ಖಂಡಿತವಾಗಿಯೂ ಇಲ್ಲ

9. ಮುಂದಿನ ಪ್ರಶ್ನೆಗಳು ತಂಬಾಕಿನ ಬಳಕೆಯ ವರ್ಜನೆ(ನಿಲ್ಲಿಸುವುದರ) ಬಗ್ಗೆ ಆಗಿವೆ

9.1 ನೀವು ಯಾವತ್ತಾದರೂ ತಂಬಾಕಿನ ಬಳಕೆಯನ್ನು ನಿಲ್ಲಿಸುವುದಾಗಿ ಯೋಚಿಸಿದ್ದೀರಾ?

Ⓐ ಹೌದು Ⓑ ಇಲ್ಲ
Ⓒ ನಾನು ತಂಬಾಕಿನ ಬಳಕೆ ಮಾಡಿಲ್ಲ

9.2 ನೀವು ತಂಬಾಕಿನ ಬಳಕೆ ವರ್ಜಿಸಲು (ನಿಲ್ಲಿಸಲು) ಪ್ರಯತ್ನಿಸಿದ್ದೀರಾ ?

Ⓐ ಹೌದು Ⓑ ಇಲ್ಲ
Ⓒ ನಾನು ತಂಬಾಕಿನ ಬಳಕೆ ಮಾಡಿಲ್ಲ

10. ಮುಂದಿನ ಪ್ರಶ್ನೆಗಳು ತಂಬಾಕು ವಿರೋಧಿ ಚಟುವಟಿಕೆಗಳ ಅರಿವಿನ ಬಗ್ಗೆ ಆಗಿರುತ್ತದೆ

10.1 ಕಳೆದ ಒಂದು ಶೈಕ್ಷಣಿಕ ವರ್ಷದಲ್ಲಿ ನಿಮ್ಮ ಶಾಲೆಯಲ್ಲಿ ತಂಬಾಕು ಮತ್ತು ತಂಬಾಕು ಉತ್ಪನ್ನಗಳ ದುಷ್ಪರಿಣಾಮಗಳ ಬಗ್ಗೆ ಯಾವುದೇ ಶೈಕ್ಷಣಿಕ ತರಗತಿಗಳನ್ನು ಏರ್ಪಡಿಸಲಾಗಿತ್ತೇ?

Ⓐ ಹೌದು Ⓑ ಇಲ್ಲ
Ⓒ ಸರಿಯಾಗಿ ತಿಳಿದಿಲ್ಲ

10.2 ನೀವು ಕೊನೆಯ ಒಂದು ವರ್ಷದಲ್ಲಿ ತಂಬಾಕು ವಿರೋಧಿ ಚಟುವಟಿಕೆಗಳಲ್ಲಿ ಭಾಗವಹಿಸಿದ್ದೀರಾ?

Ⓐ ಹೌದು
Ⓑ ಇಲ್ಲ

10.3 ನೀವು ಕಳೆದ 30 ದಿನಗಳಲ್ಲಿ ರೇಡಿಯೋ/ದೂರದರ್ಶನದಲ್ಲಿ ಎಷ್ಟು ತಂಬಾಕು ವಿರೋಧಿ ಸಂದೇಶಗಳನ್ನು ಕೇಳಿದ್ದೀರಿ/ನೋಡಿದ್ದೀರಿ?

Ⓐ ಒಂದೂ ಇಲ್ಲ Ⓑ 1 ರಿಂದ 5 ಸಂದೇಶಗಳು
Ⓒ 6 ರಿಂದ 10 ಸಂದೇಶಗಳು Ⓓ 10 ಕ್ಕಿಂತ ಜಾಸ್ತಿ ಸಂದೇಶಗಳು

10.4 ನೀವು ಕಳೆದ 30 ದಿನಗಳಲ್ಲಿ ಪೋಸ್ಟರ್‌ಗಳ ಮೇಲೆ ಎಷ್ಟು ತಂಬಾಕು ವಿರೋಧಿ ಸಂದೇಶಗಳನ್ನು ನೋಡಿದ್ದೀರಿ?

Ⓐ ಒಂದೂ ಇಲ್ಲ Ⓑ 1 ರಿಂದ 5 ಸಂದೇಶಗಳು
Ⓒ 6 ರಿಂದ 10 ಸಂದೇಶಗಳು Ⓓ 10 ಕ್ಕಿಂತ ಜಾಸ್ತಿ ಸಂದೇಶಗಳು

10.5 ನೀವು ಕಳೆದ 30 ದಿನಗಳಲ್ಲಿ ದಿನಪತ್ರಿಕೆ/ಮ್ಯಾಗಜಿನ್‌ಗಳಲ್ಲಿ ಎಷ್ಟು ತಂಬಾಕು ವಿರೋಧಿ ಸಂದೇಶಗಳನ್ನು ನೋಡಿದ್ದೀರಾ?

Ⓐ ಒಂದೂ ಇಲ್ಲ Ⓑ 1 ರಿಂದ 5 ಸಂದೇಶಗಳು
Ⓒ 6 ರಿಂದ 10 ಸಂದೇಶಗಳು Ⓓ 10 ಕ್ಕಿಂತ ಜಾಸ್ತಿ ಸಂದೇಶಗಳು
Ⓔ ನಾನು ದಿನಪತ್ರಿಕೆ/ಮ್ಯಾಗಜಿನ್‌ಗಳನ್ನು ಓದುವುದಿಲ್ಲ

11. ಮುಂದಿನ ಪ್ರಶ್ನೆಗಳು ತಂಬಾಕು ಕುರಿತಾದ ಜಾಹೀರಾತುಗಳ ಬಗ್ಗೆ ಆಗಿವೆ

11.1 ಕಳೆದ 30 ದಿನಗಳಲ್ಲಿ, ಯಾವುದಾದರು ಸಾರ್ವಜನಿಕ ಸಾರಿಗೆ (ಬಸ್ಸು, ರೈಲು, ಟ್ಯಾಕ್ಸಿ, ಇತ್ಯಾದಿ) ಯಲ್ಲಿ ತಂಬಾಕು ಉತ್ಪನ್ನಗಳ ಬಗ್ಗೆ ಜಾಹೀರಾತುಗಳನ್ನು ಗಮನಿಸಿದ್ದೀರಾ?
- Ⓐ ಹೌದು
- Ⓑ ಇಲ್ಲ

11.2 ಕಳೆದ 30 ದಿನಗಳಲ್ಲಿ, ಸಾರ್ವಜನಿಕ ಸ್ಥಳಗಳಲ್ಲಿ ಯಾವುದಾದರು ತಂಬಾಕು ಉತ್ಪನ್ನಗಳ ಕುರಿತಾದ ಜಾಹೀರಾತುಗಳನ್ನು ಗೋಡೆ ಬರಹದಲ್ಲಿ ಗಮನಿಸಿದ್ದೀರಾ?
- Ⓐ ಹೌದು
- Ⓑ ಇಲ್ಲ

11.3 ಕಳೆದ 30 ದಿನಗಳಲ್ಲಿ, ಅಂತರ್ಜಾಲ(ಇಂಟರ್ನೆಟ್)ದಲ್ಲಿ ತಂಬಾಕು ಉತ್ಪನ್ನಗಳ ಬಗ್ಗೆ ಜಾಹೀರಾತುಗಳನ್ನು ಗಮನಿಸಿದ್ದೀರಾ?
- Ⓐ ಹೌದು
- Ⓑ ಇಲ್ಲ

11.4 ಕಳೆದ 30 ದಿನಗಳಲ್ಲಿ, ರೇಡಿಯೋ/ ದೂರದರ್ಶನದಲ್ಲಿ ತಂಬಾಕು ಉತ್ಪನ್ನಗಳ ಕುರಿತಾದ ಜಾಹೀರಾತುಗಳನ್ನು ಗಮನಿಸಿದ್ದೀರಾ?
- Ⓐ ಹೌದು
- Ⓑ ಇಲ್ಲ

11.5 ಕಳೆದ 30 ದಿನಗಳಲ್ಲಿ, ದಿನಪತ್ರಿಕೆ/ಮ್ಯಾಗಜಿನ್‌ಗಳಲ್ಲಿ ತಂಬಾಕು ಉತ್ಪನ್ನಗಳ ಬಗ್ಗೆ ಜಾಹೀರಾತುಗಳನ್ನು ಗಮನಿಸಿದ್ದೀರಾ?
- Ⓐ ಹೌದು
- Ⓑ ಇಲ್ಲ

12. ಮುಂದಿನ ಪ್ರಶ್ನೆಗಳು ಚಲನಚಿತ್ರಗಳು ಮತ್ತು ಮ್ಯೂಸಿಕ್ ವೀಡಿಯೋಗಳ ಬಗ್ಗೆಯಾಗಿದೆ (ನಿಮಗೆ ಅನ್ವಯಿಸುವ ಎಲ್ಲಾ ವೃತ್ತಗಳಲ್ಲಿ ಗುರುತಿಸಿ)

12.1 ಈ ಕೆಳಗಿನ ಯಾವುದೆಲ್ಲ ಕನ್ನಡ ಚಲನಚಿತ್ರಗಳನ್ನು ವೀಕ್ಷಿಸಿದ್ದೀರಿ?
- Ⓐ ಹೆಬ್ಬುಲಿ
- Ⓑ ಅಂಜನೀಪುತ್ರ
- Ⓒ ರಾಜಕುಮಾರ
- Ⓓ ಭರ್ಜರಿ
- Ⓔ ತಾರಕ್
- Ⓕ ಚಕ್ರವರ್ತಿ
- Ⓖ ಚಮಕ್
- Ⓗ ರಾಜು ಕನ್ನಡ ಮೀಡಿಯಂ
- Ⓘ ಮೇಲ್ಕಂಡ ಯಾವುದೇ ಚಲನಚಿತ್ರ ನೋಡಿಲ್ಲ

12.2 ಈ ಕೆಳಗಿನ ಯಾವುದೆಲ್ಲ ಹಿಂದಿ ಚಲನಚಿತ್ರಗಳನ್ನು ವೀಕ್ಷಿಸಿದ್ದೀರಿ?
- Ⓐ ಟೈಗರ್ ಝಿಂದಾ ಹೈ
- Ⓑ ಗೋಲ್‌ಮಾಲ್ ಅಗೈನ್
- Ⓒ ರಈಸ್
- Ⓓ ಜುಡ್ವಾ– ೨
- Ⓔ ಟಾಯ್ಲೆಟ್ : ಏಕ್ ಪ್ರೇಮ್ ಕಥಾ
- Ⓕ ಬಾಘಿ – ೨
- Ⓖ ಸೊನು ಕೆ ಟೀಟೂ ಕೀ ಸ್ವೀಟಿ
- Ⓗ ರೈಡ್
- Ⓘ ಜಾಲೀ ಎಲ್.ಎಲ್.ಬಿ ೨
- Ⓙ ಮೇಲ್ಕಂಡ ಯಾವುದೇ ಚಲನಚಿತ್ರ ನೋಡಿಲ್ಲ

12.3 ಈ ಕೆಳಗಿನ ಯಾವುದೆಲ್ಲ ತುಳು ಚಲನಚಿತ್ರಗಳನ್ನು ವೀಕ್ಷಿಸಿದ್ದೀರಿ?
- Ⓐ ಪಿಲಿಬೈಲ್ ಯಮುನಕ್ಕ
- Ⓑ ಏಸ
- Ⓒ ಅರೆಮರ್ಲೆರ್
- Ⓓ ಅಪ್ಪೆ ಟೀಚರ್
- Ⓔ ಮೇಲ್ಕಂಡ ಯಾವುದೇ ಚಲನಚಿತ್ರ ನೋಡಿಲ್ಲ

12.4 ಈ ಕೆಳಗಿನ ಯಾವುದೆಲ್ಲ ಅನ್ಯ ಭಾಷಾ ಚಲನಚಿತ್ರಗಳನ್ನು ವೀಕ್ಷಿಸಿದ್ದೀರಿ?
- Ⓐ ಮೆರ್ಸಲ್ (ತಮಿಳು)
- Ⓑ ಡನ್‌ಕಿರ್ಕ್ (ಆಂಗ್ಲ)
- Ⓒ ಐಟಿ (ಆಂಗ್ಲ)
- Ⓓ ಮೇಲ್ಕಂಡ ಯಾವುದೇ ಚಲನಚಿತ್ರ ನೋಡಿಲ್ಲ

12.5ಈ ಕೆಳಗಿನ ಯಾವುದೆಲ್ಲ ಕನ್ನಡ ಮ್ಯೂಸಿಕ್ ವೀಡಿಯೋಗಳನ್ನು ನೋಡಿದ್ದೀರಿ?
- Ⓐ ಟಕಿಲ
- Ⓑ ಅಪ್ಪಾ ಐ ಲವ್ ಯೂ ಪಾ
- Ⓒ ಚೊಕಲೇಟ್ ಗರ್ಲ್
- Ⓓ ದಮ್ ಮಾರೊ ದಮ್
- Ⓔ ಮೇಲ್ಕಂಡ ಯಾವುದೇ ಮ್ಯೂಸಿಕ್ ವೀಡಿಯೋಗಳನ್ನು ನೋಡಿಲ್ಲ

12.6 ಈ ಕೆಳಗಿನ ಯಾವುದೆಲ್ಲ ಅನ್ಯ ಭಾಷಾ ಮ್ಯೂಸಿಕ್ ವೀಡಿಯೋಗಳನ್ನು ನೋಡಿದ್ದೀರಿ?
- Ⓐ ಮೀಸಯ ಮುರುಕ್ಕು (ತಮಿಳು)
- Ⓑ ಸೀಟಿ ಮಾರ್ (ತೆಲುಗು)
- Ⓒ ಗುಲೆಬಾ ಗುಲೆಬಾ (ತಮಿಳು)
- Ⓓ ಮೇಲ್ಕಂಡ ಯಾವುದೇ ಮ್ಯೂಸಿಕ್ ವೀಡಿಯೋವನ್ನು ನೋಡಿಲ್ಲ

12.7 ನೀವು ಚಲನಚಿತ್ರ/ಮ್ಯೂಸಿಕ್ ವೀಡಿಯೋಗಳನ್ನು ವೀಕ್ಷಿಸುವಾಗ ಯಾವುದೇ ರೀತಿಯ ತಂಬಾಕು ವಿರೋಧಿ ಸಂದೇಶಗಳನ್ನು ಗಮನಿಸಿದ್ದೀರಾ ?
- Ⓐ ಹೌದು
- Ⓑ ಇಲ್ಲ

12.8 ನೀವು ನೋಡಿದ ಚಲನಚಿತ್ರ/ಮ್ಯೂಸಿಕ್ ವೀಡಿಯೋಗಳಲ್ಲಿ ನಟ/ನಟಿಯರು ಧೂಮಪಾನ ಮಾಡುವುದನ್ನು ಗಮನಿಸಿದ್ದೀರಾ ?
- Ⓐ ಹೌದು
- Ⓑ ಇಲ್ಲ

12.9 ನೀವು ನೋಡಿದ ಚಲನಚಿತ್ರ/ಮ್ಯೂಸಿಕ್ ವೀಡಿಯೋಗಳಲ್ಲಿ ನಟ/ನಟಿಯರು ಹೊಗೆರಹಿತ ತಂಬಾಕಿನ ಬಳಕೆ ಮಾಡುವುದನ್ನು ಗಮನಿಸಿದ್ದೀರಾ ?
- Ⓐ ಹೌದು
- Ⓑ ಇಲ್ಲ

13. ಈ ಕೆಳಗಿನ ವಾಕ್ಯಗಳನ್ನು ಓದಿ ಮತ್ತು ನಿಮ್ಮ ಮನೋಭಾವನೆಯನ್ನು ತಿಳಿಸಿ

13.1 ನನ್ನ ಪ್ರಕಾರ ನಾನು ಏನಾದರೂ ಮಾಡುವುದಾದರೆ, ನಾನು ಯಾವುದೇ ನಿಯಮಗಳನ್ನು ಪರಿಗಣಿಸುವುದಿಲ್ಲ
- Ⓐ ಯಾವತ್ತೂ ಇಲ್ಲ
- Ⓑ ಕೆಲವೊಮ್ಮೆ
- Ⓒ ಯಾವಾಗಲೂ

13.2 ನನ್ನ ತಂದೆ ತಾಯಿಗೆ ಇಚ್ಛೆ ಇಲ್ಲದಿದ್ದರೂ, ನಾನು ಆ ಕೆಲಸವನ್ನು ಮಾಡುತ್ತೇನೆ
- Ⓐ ಯಾವತ್ತೂ ಇಲ್ಲ
- Ⓑ ಕೆಲವೊಮ್ಮೆ
- Ⓒ ಯಾವಾಗಲೂ

13.3 ನಾನು ಶಾಲೆ, ಕೆಲಸ ಅಥವಾ ಇತರ ಸ್ಥಳಗಳಲ್ಲಿ ತೊಂದರೆಗೆ ಸಿಲುಕಿ ಕೊಳ್ಳುತ್ತೇನೆ
- Ⓐ ಯಾವತ್ತೂ ಇಲ್ಲ
- Ⓑ ಕೆಲವೊಮ್ಮೆ
- Ⓒ ಯಾವಾಗಲೂ

14. ಈ ಮುಂದಿನ ಪ್ರಶ್ನೆಗಳು ನಿಮ್ಮ ಕುರಿತು ಮತ್ತು ಪರೀಕ್ಷೆಯಲ್ಲಿ ನಿಮ್ಮ ಸಾಧನೆ ಕುರಿತಾಗಿದೆ

14.1 ಈ ವಾಕ್ಯವನ್ನು ಓದಿ ಮತ್ತು ನಿಮಗೆ ಸರಿ ಅನಿಸಿದ ಆಯ್ಕೆಯನ್ನು ಗುರುತಿಸಿ.
"ನನ್ನ ಆಲೋಚನೆ ಪ್ರಕಾರ ನನಗೆ ಹೆಚ್ಚಿನ ಸ್ವಾಭಿಮಾನವಿದೆ"
- Ⓐ ದೃಢವಾಗಿ ಸಮ್ಮತಿಸುತ್ತೇನೆ
- Ⓑ ಸಮ್ಮತಿಸುತ್ತೇನೆ
- Ⓒ ಸಮ್ಮತಿ ಇಲ್ಲ, ಅಸಮ್ಮತಿಯೂ ಇಲ್ಲ
- Ⓓ ಅಸಮ್ಮತಿಸುತ್ತೇನೆ
- Ⓔ ದೃಢವಾಗಿ ಅಸಮ್ಮತಿಸುತ್ತೇನೆ

14.2 ಕಳೆದ ವಾರ್ಷಿಕ ಪರೀಕ್ಷೆಯಲ್ಲಿ ನಿಮ್ಮ ಸಾಧನೆಯ ಗುಣಮಟ್ಟವನ್ನು ತಿಳಿಸಿ
- Ⓐ ಅತ್ಯುತ್ತಮ
- Ⓑ ಉತ್ತಮ
- Ⓒ ಸಾಧಾರಣ
- Ⓓ ಸಾಧಾರಣಕ್ಕಿಂತ ಕಡಿಮೆ

15. ಕೊನೆಯ ಪ್ರಶ್ನೆ ನಿಮ್ಮ ಮನೆಗೆ ಸಂಬಂಧಿಸಿದ್ದಾಗಿರುತ್ತದೆ

15.1 ನಿಮ್ಮ ಮನೆಯಲ್ಲಿ ಇರುವ ವಸ್ತುಗಳು ಅಥವಾ ನಿಮ್ಮ ಮನೆಯಲ್ಲಿ ವಾಸವಾಗಿರುವ ವ್ಯಕ್ತಿಗಳಿಗೆ ಸೇರಿದ ವಸ್ತುಗಳಿಗೆ ಗುರುತು ಹಾಕಿ :
- Ⓐ ವಿದ್ಯುತ್ (ಕರೆಂಟ್)
- Ⓑ ಫ್ಲಷ್ ಇರುವ ಶೌಚಾಲಯ
- Ⓒ ಕಾರು
- Ⓓ ದ್ವಿಚಕ್ರ ವಾಹನ
- Ⓔ ಟಿ.ವಿ
- Ⓕ ಫ್ರಿಡ್ಜ್
- Ⓖ ವಾಷಿಂಗ್ ಮೆಷಿನ್
- Ⓗ ಲ್ಯಾಂಡ್ ಲೈನ್ ಫೋನ್
- Ⓘ ಮೊಬೈಲ್ ಫೋನ್
- Ⓙ ರೇಡಿಯೋ

ಈ ಪ್ರಶ್ನಾವಳಿಯನ್ನು ಭರ್ತಿಗೊಳಿಸಿದ್ದಕ್ಕಾಗಿ ಧನ್ಯವಾದಗಳು

ಸಂಶೋಧನಾ ಸಹಾಯಕರ ಸಹಿ
