# Supplementary material for: Prospective cohort study of exposure to tobacco imagery in popular films and smoking uptake among children in southern India
Source: PLoS One. 2021 Aug 5;16(8):e0253593. doi: 10.1371/journal.pone.0253593 (PMC8341541; doi:10.1371/journal.pone.0253593)
Supplement: Attachment — Submitted filename: Response to Reviewers.docx [file pone.0253593.s003.docx]

**Journal Requirements:**

1. Please ensure that your manuscript meets PLOS ONE's style requirements, including those for file naming. The PLOS ONE style templates can be found at https://journals.plos.org/plosone/s/file?id=wjVg/PLOSOne_formatting_sample_main_body.pdf and https://journals.plos.org/plosone/s/file?id=ba62/PLOSOne_formatting_sample_title_authors_affiliations.pdf

**Authors Reply:** The authors have reviewed the manuscript thoroughly to ensure it meets the PLOS ONE’s style requirement.

2. Please include additional information regarding the survey or questionnaire used in the study and ensure that you have provided sufficient details that others could replicate the analyses.

For instance, if you developed a questionnaire as part of this study and it is not under a copyright more restrictive than CC-BY, please include a copy, in both the original language and English, as Supporting Information.

**Authors Reply:** The questionnaire in the original language and in English has been included as supporting information.

3. We note that you have indicated that data from this study are available upon request. PLOS only allows data to be available upon request if there are legal or ethical restrictions on sharing data publicly. For information on unacceptable data access restrictions, please see http://journals.plos.org/plosone/s/data-availability#loc-unacceptable-data-access-restrictions.

In your revised cover letter, please address the following prompts:

a) If there are ethical or legal restrictions on sharing a de-identified data set, please explain them in detail (e.g., data contain potentially identifying or sensitive patient information) and who has imposed them (e.g., an ethics committee). Please also provide contact information for a data access committee, ethics committee, or other institutional body to which data requests may be sent.

b) If there are no restrictions, please upload the minimal anonymized data set necessary to replicate your study findings as either Supporting Information files or to a stable, public repository and provide us with the relevant URLs, DOIs, or accession numbers. Please see http://www.bmj.com/content/340/bmj.c181.long for guidelines on how to de-identify and prepare clinical data for publication. For a list of acceptable repositories, please see http://journals.plos.org/plosone/s/data-availability#loc-recommended-repositories.

We will update your Data Availability statement on your behalf to reflect the information you provide. The data may

**Authors Reply**: The data will be made available on reasonable request as per the guidelines of institutional ethics committee by contacting the below mentioned email address by researchers who meet the criteria by access to confidential data. (Email: [mrcuk.antitobacco@manipal.edu](mailto:mrcuk.antitobacco@manipal.edu))

4. Thank you for stating the following financial disclosure: 'The funders had no role in study design, data collection and analysis, decision to publish, or preparation of the manuscript.'

At this time, please address the following queries:

Please clarify the sources of funding (financial or material support) for your study. List the grants or organizations that supported your study, including funding received from your institution.

State what role the funders took in the study. If the funders had no role in your study, please state: “The funders had no role in study design, data collection and analysis, decision to publish, or preparation of the manuscript.”

If any authors received a salary from any of your funders, please state which authors and which funders.

If you did not receive any funding for this study, please state: “The authors received no specific funding for this work.”

Please include your amended statements within your cover letter; we will change the online submission form on your behalf.

**Authors Reply:** The authors are fulltime employees of their respective institutions and draw incentives to compensate for the time through the grant. Dr. Rohit Bhagawath is the Social Scientist in the project and gets his salary from the project and not from the host institution.

5. We note that the grant information you provided in the ‘Funding Information’ and ‘Financial Disclosure’ sections do not match.

When you resubmit, please ensure that you provide the correct grant numbers for the awards you received for your study in the ‘Funding Information’ section.

**Authors Reply:** The grant numbers received for our study in the ‘Funding Information’ section has been verified.

**Reviewers' comments:**

**Reviewer's Responses to Questions**

Comments to the Author

1. Is the manuscript technically sound, and do the data support the conclusions?

The manuscript must describe a technically sound piece of scientific research with data that supports the conclusions. Experiments must have been conducted rigorously, with appropriate controls, replication, and sample sizes. The conclusions must be drawn appropriately based on the data presented.

Reviewer #1: Yes

Reviewer #2: No

**Authors Reply:** In this revised submission we have attempted to address Reviewer #2’s concerns in these areas.

2. Has the statistical analysis been performed appropriately and rigorously?

Reviewer #1: Yes

Reviewer #2: No

**Authors Reply:** As above, we have endeavoured to resolve all of Reviewer #2’s concerns over our analysis.

3. Have the authors made all data underlying the findings in their manuscript fully available?

The PLOS Data policy requires authors to make all data underlying the findings described in their manuscript fully available without restriction, with rare exception (please refer to the Data Availability Statement in the manuscript PDF file). The data should be provided as part of the manuscript or its supporting information, or deposited to a public repository. For example, in addition to summary statistics, the data points behind means, medians and variance measures should be available. If there are restrictions on publicly sharing data—e.g. participant privacy or use of data from a third party—those must be specified.

Reviewer #1: Yes

Reviewer #2: Yes

**Authors Reply:** None necessary.

4. Is the manuscript presented in an intelligible fashion and written in standard English?

PLOS ONE does not copyedit accepted manuscripts, so the language in submitted articles must be clear, correct, and unambiguous. Any typographical or grammatical errors should be corrected at revision, so please note any specific errors here.

Reviewer #1: Yes

Reviewer #2: No

**Authors Reply:** Again, we have endeavoured to resolve all of Reviewer #2’s concerns over intelligibility and use of English.

5. Review Comments to the Author

Please use the space provided to explain your answers to the questions above. You may also include additional comments for the author, including concerns about dual publication, research ethics, or publication ethics. (Please upload your review as an attachment if it exceeds 20,000 characters)

Reviewer #1: It was a pleasure to read this well written paper. The researchers have a large data set that assesses the risk of taking up smoking over two years by analysing a variety of home and exposure to smoking in films. I have a few comments that will improve the manuscript.

1. as PLOS One has no word count more description of the explanatory variables is needed rather than referring to a previous publication. Research papers should, as much as possible, stand alone. I realise that the less well explained variables are the control variables but the same level of detail is needed to assist in interpreting the tables.

**Authors reply:** Thank you for the suggestion. We have now provided a more complete description of all the variables, but to maintain the readability of the paper have provided these in an Annexe document.

2. I’d be happier seeing all the variables in the final model to see how adding the film exposure variables affects the control variables. This is a matter of convention and in environmental health the convention is to report them all. This is, essentially a report about the impact of environmental factors.

**Authors reply:** We have now added additional model to both the tables that includes all the variables irrespective of their significance along with film tobacco imagery.

3. Be more honest in the discussion as only 1 of 5 measures of film exposure was statistically significant. Given the magnitude of the home effects it is surprising that anything from the wider environment is statistically significant. Just don’t over-egg the finding.

**Authors reply:** We have again analysed the data creating another model as described above and the results have been provided in both the tables.

4. The discussion should bring in more literature about the how home and wider social factors intersect. There will be research about this in sociological literature.

**Authors reply:** Thank you for the suggestion. We have now added a paragraph in the discussion to address this concern of the reviewer.

5. There does not seem to be a section in the discussion on limitations of the study. One of these is the relatively small number of students who took up smoking. Was there a sample size calculation in the original research plan?

I hope you are able to find funding to continue following up this cohort.

**Authors reply:**

We have added the below calculation in methods section as we had proposed in the study protocol in the methods section.

Reviewer #2:

6. Unfortunately, I still believe that the presentation of the study findings remains incomprehensible for the general reader. Table 1 should show percentages along with frequencies. Table 3 remain incomprehensible for the general reader.

**Authors reply:** Thank you for the suggestion. We have simplified the language wherever possible. We have included percentages along with frequencies in Table 1. We have provided explanation for all the variables in the Annexure attached as supplementary file.

6. PLOS authors have the option to publish the peer review history of their article (what does this mean?). If published, this will include your full peer review and any attached files.

If you choose “no”, your identity will remain anonymous but your review may still be made public.

Do you want your identity to be public for this peer review? For information about this choice, including consent withdrawal, please see our Privacy Policy.

Reviewer #1: Yes: Professor Shona J. Kelly

Reviewer #2: Yes: Elpidoforos S. Soteriades

[NOTE: If reviewer comments were submitted as an attachment file, they will be attached to this email and accessible via the submission site. Please log into your account, locate the manuscript record, and check for the action link "View Attachments". If this link does not appear, there are no attachment files.]

While revising your submission, please upload your figure files to the Preflight Analysis and Conversion Engine (PACE) digital diagnostic tool, https://pacev2.apexcovantage.com/. PACE helps ensure that figures meet PLOS requirements. To use PACE, you must first register as a user. Registration is free. Then, login and navigate to the UPLOAD tab, where you will find detailed instructions on how to use the tool. If you encounter any issues or have any questions when using PACE, please email PLOS at figures@plos.org. Please note that Supporting Information files do not need this step.

In compliance with data protection regulations, you may request that we remove your personal registration details at any time. (Remove my information/details). Please contact the publication office if you have any questions.
